# ZIP Code-Level Estimation of Air Quality and Health Risk Due to Particulate Matter Pollution in New York City

**DOI:** 10.1021/acs.est.1c07325

**Published:** 2022-04-27

**Authors:** Komal Shukla, Catherine Seppanen, Brian Naess, Charles Chang, David Cooley, Andreas Maier, Frank Divita, Masha Pitiranggon, Sarah Johnson, Kazuhiko Ito, Saravanan Arunachalam

**Affiliations:** † Institute for the Environment, The University of North Carolina at Chapel Hill, Chapel Hill, North Carolina 27599, United States; ‡ Abt Associates, Durham, North Carolina 27703, United States; § New York City Department of Health and Mental Hygiene, Bureau of Environmental Surveillance and Policy, New York, New York 10013, United States

**Keywords:** particulate matter, emissions, health benefits, New York City, policy research, ZIP code, ZAPPA, COBRA, C-TOOLS

## Abstract

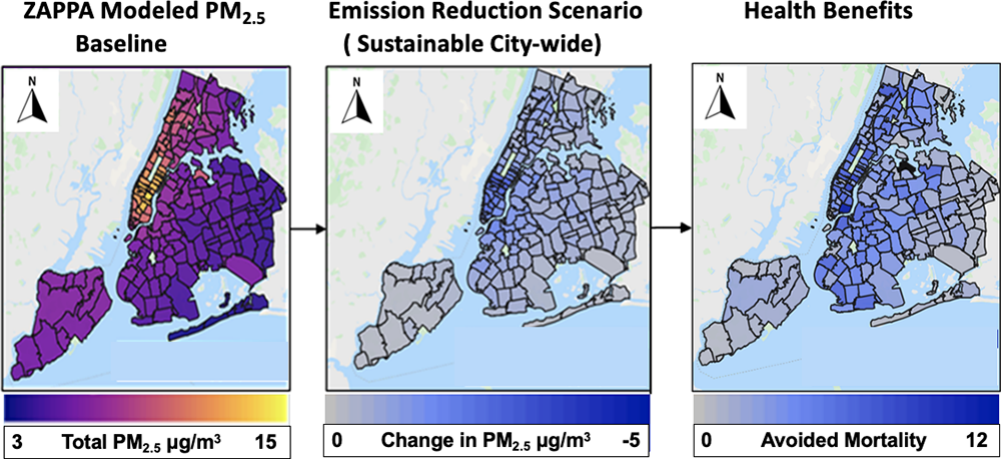

Exposure to PM_2.5_ is associated with hundreds of premature mortalities every year in New York City (NYC). Current air quality and health impact assessment tools provide county-wide estimates but are inadequate for assessing health benefits at neighborhood scales, especially for evaluating policy options related to energy efficiency or climate goals. We developed a new ZIP Code-Level Air Pollution Policy Assessment (ZAPPA) tool for NYC by integrating two reduced form models—Community Air Quality Tools (C-TOOLS) and the Co-Benefits Risk Assessment Health Impacts Screening and Mapping Tool (COBRA)—that propagate emissions changes to estimate air pollution exposures and health benefits. ZAPPA leverages custom higher resolution inputs for emissions, health incidences, and population. It, then, enables rapid policy evaluation with localized ZIP code tabulation area (ZCTA)-level analysis of potential health and monetary benefits stemming from air quality management decisions. We evaluated the modeled 2016 PM_2.5_ values against observed values at EPA and NYCCAS monitors, finding good model performance (FAC2, 1; NMSE, 0.05). We, then, applied ZAPPA to assess PM_2.5_ reduction-related health benefits from five illustrative policy scenarios in NYC focused on (1) commercial cooking, (2) residential and commercial building fuel regulations, (3) fleet electrification, (4) congestion pricing in Manhattan, and (5) these four combined as a “citywide sustainable policy implementation” scenario. The citywide scenario estimates an average reduction in PM_2.5_ of 0.9 μg/m^3^. This change translates to avoiding 210–475 deaths, 340 asthma emergency department visits, and monetized health benefits worth $2B to $5B annually, with significant variation across NYC’s 192 ZCTAs. ZCTA-level assessments can help prioritize interventions in neighborhoods that would see the most health benefits from air pollution reduction. ZAPPA can provide quantitative insights on health and monetary benefits for future sustainability policy development in NYC.

## Abstract 2

A new high resolution policy assessment tool has been developed to quantify air quality and health benefits of emissions reductions in NYC.

## Introduction

Fine particulate matter (PM_2.5_) (particulate matter of size less than 2.5 μm) has been associated in multiple epidemiological studies^1−4^ with adverse health outcomes, such as cardiovascular and respiratory disease, neurological impacts, lung cancer,^3^ and premature mortality.^5^ Recent estimates show a global burden of 8.7 million premature mortalities due to ambient PM_2.5_ from fossil fuel combustion.^6^ Despite steady improvements in air quality in the United States because of the Clean Air Act Amendments and other state and local emission control measures, air pollution is estimated to account for 5–10% of annual deaths nationally, with approximately 88 400 deaths attributed to PM_2.5_ pollution.^7,8^

PM_2.5_ is a complex pollutant that includes both primary (directly emitted from combustion sources, such as vehicle and power plants, road dust, and cooking) and secondary (formed by various physical and chemical processes due to the interaction between gaseous precursors (NOx, SOx, NH_3_, VOC) and aqueous chemistry)^9^ components. To reduce PM_2.5_ levels in ambient air to protect public health, continued reductions in both primary PM and precursor gases that form secondary PM are essential. Thus, to estimate total PM_2.5_ that cause adverse health impacts in humans, one must adequately characterize both primary and secondary components to study PM_2.5_ health impacts.^10^

New York City (NYC), the densest city in the United States with an area of 783 km^2^ and a population of more than 8.41 million (population density of 27 346 people/sq-km), continues to experience major impacts from PM_2.5_.^11,12^ NYC has an ever-increasing energy demand in all its emissions sectors (buildings, on-road, and power generation). Despite multiple policies addressing fuel switching and technology changes, emissions remain high enough to impact the city’s population. In the past decade, multiple studies focused on PM_2.5_ pollution in NYC, including concentration trends^11−15,22^ source apportionment,^16,17^ health burden,^18−23^ and emission control.^25,26^ Despite declines in PM_2.5_ concentrations (New York City Community Air Survey (NYCCAS)^27^ 2019 and U.S. EPA monitoring observations from 2011 to 2020), the health burden attributed to current ambient levels of PM_2.5_ is still high enough to be of concern.^21^ The NYC Department of Health and Mental Hygiene estimates that annually (2015–2017 average) almost 2000 premature deaths and more than 6500 emergency department (ED) visits and hospitalizations can be attributed to PM_2.5_ emissions in the region from multiple sources.^28^ Fine particulate emission sources in NYC include fuel combustion for heating and hot water in commercial and residential buildings, commercial cooking, on-road vehicles, and off-highway construction and freight distribution. Traffic emissions from the New York Metropolitan Statistical Area (NYMSA) were estimated to have caused ∼1800 premature deaths due to exposure to PM_2.5_ and O_3_, with 20% due to emissions from medium-duty trucks (MDT) and 17% due to the heavy-duty trucks (HDT) sector.^11^ Jin et al.^29^ used multiple estimates of PM_2.5_ exposures for New York state from varying techniques (satellite remote sensing, air quality modeling, land use regression modeling, etc.) and assess gains in air pollution-related health benefits for a decadal period and state a 28% uncertainty because of choice of different techniques for estimating air pollution levels. Johnson et al.,^25^ however, use a comprehensive air quality model to assess the PM_2.5_ related health benefits of various policy scenarios including NYC’s Roadmap to 80 × 50. But this study highlights the inability to perform rapid turn-around of multiple scenarios because of the complex modeling framework and the associated computational burden.

NYC is demographically and geographically diverse with about 192 (fused in some cases to facilitate stable health rate estimates) ZIP Code Tabulation Areas (ZCTAs) established by the U.S. Census Bureau within its five counties. Each ZCTA has varying population demographics and emission sources, and its residents experience unique impacts from pollution exposure. Health and monetary cobenefits from pollution reduction vary among ZCTAs because of these distinct population characteristics and differential impacts of policy at the local scale.^21,25,30^ The areal extent of the ZCTAs vary from an average of 0.8 sq km in densely populated Manhattan County to an average of 10.8 sq km in Richmond County of NYC (see Table S7). Typically, PM_2.5_ studies are performed at coarse resolution (grids of 36 km × 36 km, 12 km × 12 km or sometimes county level), but NYC county-level assessment inadequately characterizes this known variability in air pollution and its impacts within the city. To address these limitations, new high resolution modeling approaches have been implemented,^21,25,30^ including utilizing satellite-derived Aerosol Optical Depth,^24,31^ air quality models like the Community Multiscale Air Quality (CMAQ) model,^32,33^ neural network forecast models,^34^ and statistical Bayesian models.^35^ Chang et al. (2017)^36^ developed a hybrid modeling approach that characterized the near-road impacts of traffic-related primary PM_2.5_ at a very high resolution and demonstrated that the hybrid approach estimated 24% more on-road PM_2.5_-related premature mortality than just using CMAQ. But the long lag time of this computationally intensive modeling prohibits iterative policy assessment and rapidly assessing responses to emerging policy goals. Studies, such as Clougherty et al. (land-use regression (LUR))^37^ and Huang et al. (LUR plus temporal predictors),^38^ have predicted PM_2.5_ concentration at finer resolution in NYC using nonregulatory observations, such as those from NYCCAS and satellite retrievals. However, these approaches cannot be feasibly integrated in a tool for rapid assessment of changes in specific input drivers for estimating potential benefits of policy outcomes. There is thus a need for a tool that is computationally less resource intensive, that includes highly resolved local emissions, has the ability to characterize and visualize potential changes in air quality levels, and quantify health benefits at a high resolution in a rapid turnaround manner. More importantly, such a tool needs to be enabled for easier use by policy makers without the comprehensive technical expertise that is needed for using detailed chemistry-transport models.

This study describes the development of the new ZIP Code Air Pollution Policy Assessment (ZAPPA) tool, that integrates two reduced-form modeling systems to support the assessment of change at the ZCTA level in adverse health events and associated costs attributable to change in PM_2.5_ emissions. The tool integrates and expands two existing models: the University of North Carolina at Chapel Hill’s C-TOOLS (Community Air Quality Tools) and the Environmental Protection Agency (EPA’s) Co-Benefits Risk Assessment (COBRA) screening model to provide hyper-local health impact assessment (HIA). ZAPPA’s unique capability to associate ZCTA-specific population and health incidences with its PM_2.5_ concentration increases its accuracy in estimating the health burden. It can model both baseline PM_2.5_ concentrations and quantify the health benefits of changes in emissions in individual sectors in a subset of the city or the entire city within a few hours, and empowers the user to perform interpretive analyses easily and generate tables and maps.

We evaluated ZAPPA’s air pollution estimates against air pollution monitoring data in New York city and used ZAPPA to assess health benefits of five illustrative policy scenarios.

## Methods

### ZAPPA Development

The web-based ZAPPA tool (https://treehug-app.its.unc.edu/zappa/) integrates C-TOOLS and COBRA. C-TOOLS^39,40^ [Community Air Quality Tools (both C-LINE and C-PORT)] is a suite of local-scale air quality screening tools previously developed and applied in multiple studies.^40,41^ In ZAPPA, C-TOOLS uses meteorological inputs from LaGuardia Airport and NYC-specific annual emissions^42^ from the year 2016 for dispersion.

**Figure 1:**
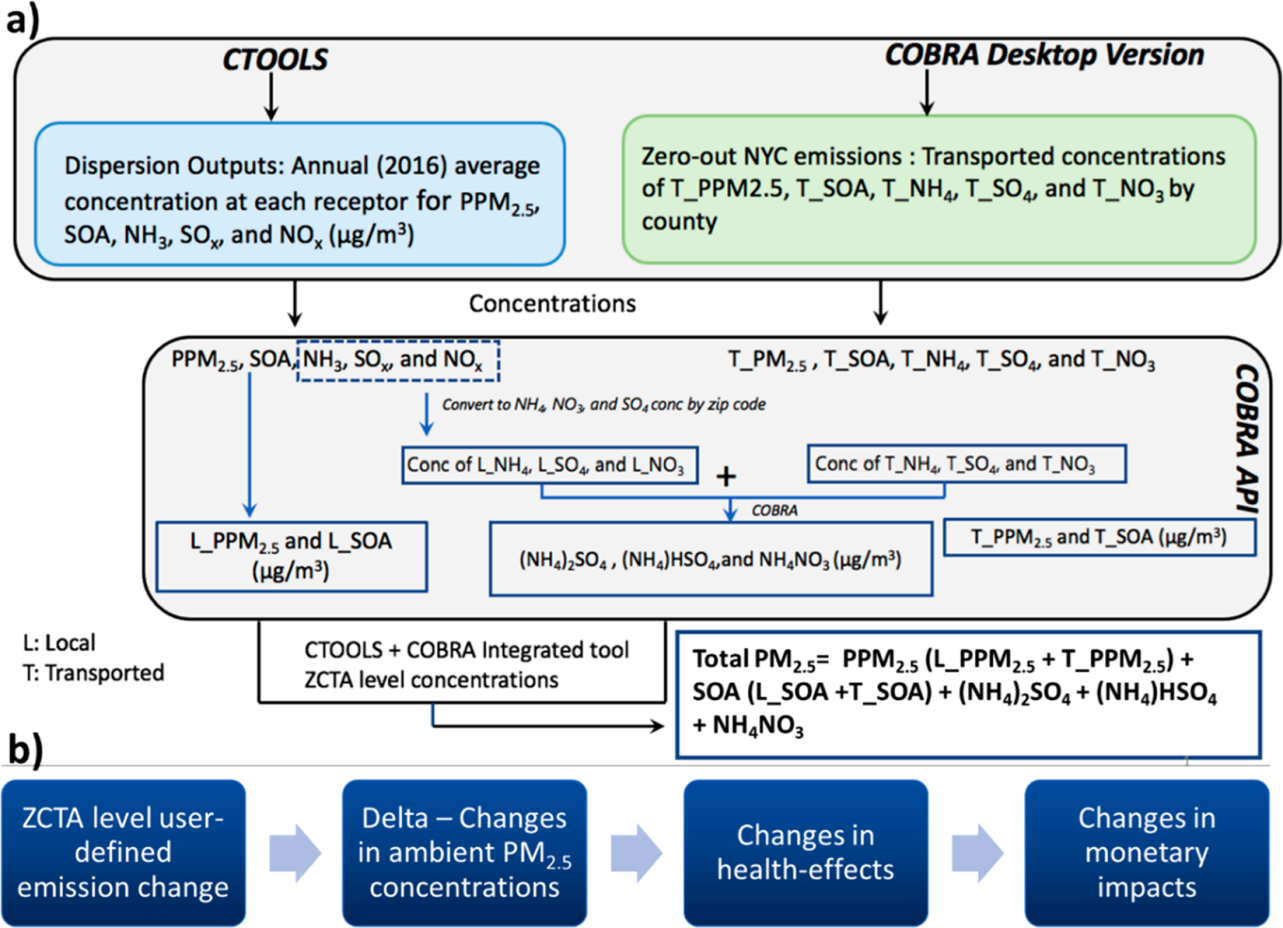
(a) ZAPPA-based total PM_2.5_ estimation and (b) ZAPPA modeling framework overview.

The Co-Benefits Risk Assessment (COBRA) model estimates total PM_2.5_ concentrations, a range of health impacts due to changes in PM_2.5_, including premature mortality, and monetized health impacts at a county-scale resolution for the entire U.S.^43^ We adapted COBRA to produce similar estimates at the ZCTA level for NYC, taking advantage of the high-resolution dispersion outputs from C-TOOLS. After total PM_2.5_ is calculated for a given emissions scenario (Figure 1), the change in PM_2.5_ from baseline is computed for any “*what if*” scenario.

The ZAPPA tool allows a user to make changes in any tier-based emission among the ten tier categories (tiers in 1a). These emissions can also be modified at the subtier (tier 2 and tier 3; fuel usage and technology) level, where the user can specify emission change in tons/year or % change. The user can select any region (ZCTA/borough/city) and modify these values for a scenario. For example, in designing a sustainable transportation scenario, the user can make changes in vehicular activity in Annual Average Daily Traffic (AADT) and fuel mix for vehicles to represent electrification of certain classes of vehicles, which would lead to less gasoline/diesel emissions. This would then translate to changes in total PM_2.5_ after applying the specific emission change, which then would be further translated to health benefits and monetized values of health benefits achieved by the change in total PM_2.5_ compared to baseline concentrations. More details are in the Supporting Information (section A1).

### Emissions Processing

**Figure 2:**
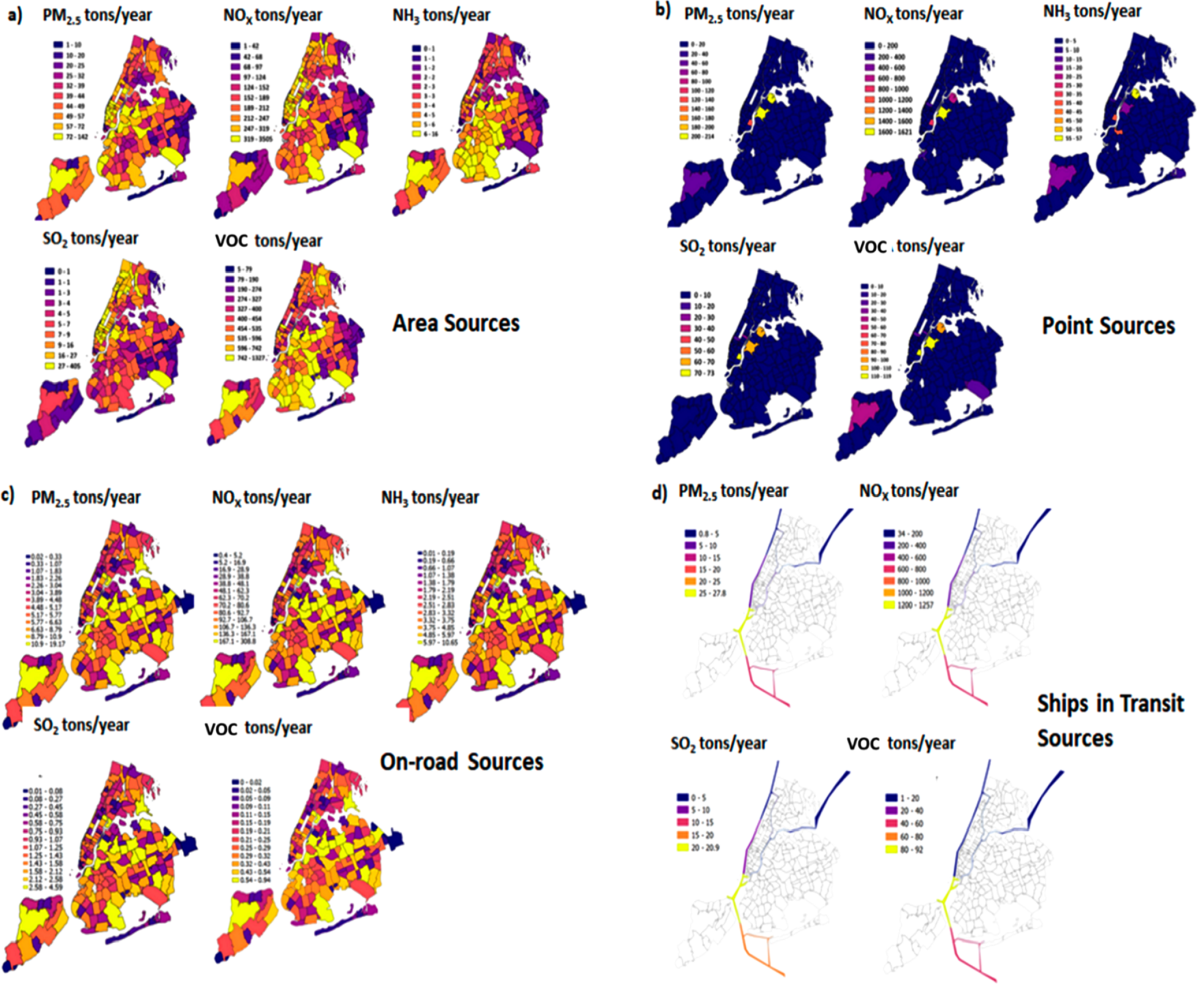
ZCTA-level PM2.5 emissions from (a) area sources, (b) point sources, (c) line sources for on-road, and (d) line sources for ships in transit (SIT).

Emissions for NYC are derived from the NEI 2016v1 emissions modeling platform,^44^ which is a product of the National Emissions Inventory Collaborative and includes a full suite of base year (2016) emissions. These are augmented for ZAPPA with some adjustments or replacements whenever more accurate or higher resolution data specific to NYC was available, such as emissions from building boilers, commercial cooking, and link-level traffic information.^25,45^ The overall emissions for PM_2.5_, NOx, SO_2_, VOC, and NH_3_ from NYC have been divided into three major groups: (1) all area emissions from ground-level, excluding the following two source categories; (2) point emissions from power generation; and (3) line emissions from on-road vehicles and ships in transit, based on the dispersion algorithms that C-TOOLS supports (Figures 2 and S1–S3). Emissions estimates by tier at the source classification code (SCC) level are assigned to each of the ZCTA polygons using the Sparse Matrix Operator Kernel Emissions (SMOKE)^46^ by applying spatial surrogates (for area sources). More information on emission types and years (Tables S1–S6) and web integration process (sections A1–A6) are detailed in the SI. We estimated secondary organic aerosols (SOA) by first scaling source-specific estimates of VOCs to SOA (see COBRA manual), and then dispersing the SOA-equivalent of VOC emissions in C-TOOLS. The VOC emissions are anthropogenic and do not include biogenic sources.

### Meteorological Data

ZAPPA modeling uses annual meteorological inputs from the LaGuardia (LGA) station (NY14732 NYC LGA; 11 km from center of NYC domain) processed through AERMET, AERMOD’s meteorological preprocessor.^47^ We increased roughness length by 0.25 m (against an urban roughness length standard 1.0 m for the rest of the city) in the Manhattan borough (New York county) to include the effect of building downwash phenomenon.^48^ Emissions sources are modeled using 100 representative hours for the year and applying weights for LGA 2016 (using the Meteorologically Weighted Averaging for Risk and Exposure (METARE) approach^36^) for computing annual average concentrations.

### Receptor Network

ZCTA-level polygons are the building blocks for the modeling in this study. 237 polygons representing 192 ZCTAs were used to allocate emissions, and a receptor network was created based on the geometry. For ZCTA polygons with an area between 100 000 and 500 000 sq feet (about the area of a Manhattan city block), a single receptor was placed at the centroid of the polygon. For larger ZCTA polygons, we used an approach based on *k*-means clustering, which first divides the ZCTA into ten smaller Voronoi polygons, while preserving the shape of the original polygon. Receptors are then placed at the centroid of each of the ten polygons, yielding a total of 1990 receptors that provide a high-resolution spatial representation of the ZCTA polygons. Receptor height is taken as 1.8 m based on the average breathing height and height of monitors.

### Model Evaluation

We evaluated individual receptor-based total PM_2.5_ estimates using a set of statistical performance measures suggested by the literature^49^ (Table 1) against 16 EPA Air Quality System (AQS) monitoring locations in NYC.

**Table 1:** Paired-Wise Comparison of PM_2.5_ (All in μg/m^3^) at Observation Locations and Model Evaluation Using Statistical Measures^49^

network	site name	NYC borough	ZCTA ID	observed	modeled	bias (modeled-observed)	% difference
AQS	JHS 126	Brooklyn	11222	7.87	7.99	0.12	1.5%
AQS	PS 274	Brooklyn	11221	6.42	6.69	0.27	4.0%
AQS	PS 314	Brooklyn	11220	6.42	7.90	1.48	18.7%
AQS	CCNY	Manhattan	10031	8.14	10.17	2.03	19.9%
AQS	Division Street	Manhattan	10038	8.01	10.58	2.57	24.2%
AQS	Intermediate School 143	Manhattan	10033	8.50	11.26	2.76	24.5%
AQS	IS 45	Manhattan	10035	7.32	9.34	2.02	21.6%
AQS	PS 19	Manhattan	10009	8.23	11.54	3.31	28.7%
AQS	Freshkills West	Staten island	10314	7.33	6.37	–0.96	–15%
AQS	Richmond Post Office	Staten island	10302	6.76	6.80	0.04	0.5%
AQS	IS 52	Bronx	10459	6.13	7.86	1.73	22.0%
AQS	IS 74	Bronx	10474	7.12	7.14	0.02	0.2%
AQS	Morrisania	Bronx	10452	6.62	10.05	3.43	34.1%
AQS	Pfizer Lab Site	Bronx	10467	8.19	8.67	0.48	5.5%
AQS	Maspeth Library	Queens	11378	6.46	6.18	–0.28	–4.5%
AQS	Queens College 2	Queens	11367	6.60	5.89	–0.71	–12.0%
NYCCAS	Staten Island	Richmond	10306	6.12	6.33	0.21	3.3%
NYCCAS	Queens	Queens	11367	6.59	5.52	–1.07	–19.3%
NYCCAS	New York	New York	10026	6.77	10.89	4.12	37.8%

model evaluation using statistical measures			
model evaluation statistics	results	acceptance criteria	satisfies
FB (fractional bias)	3.36%	within ±30%	yes
MG (geometric mean bias)	0.88	closer to ±1	yes
NMSE (normalized mean square error)	0.05	closer to 0	yes
VG (geometric variance)	1.04	ranges between ±1	yes
FAC2 (fraction of data within a factor of 2 of observations)	1	closer to ±1	yes
MFB (mean fractional bias)	9.10%	within ±30%	yes

### Health Benefits Assessment

The change in ambient PM_2.5_ concentration in each scenario is used to estimate health impacts using the COBRA Applications Programming Interface (API). COBRA defaults were updated with NYC-specific ZCTA-level population data, ZCTA-level baseline health incidence, baseline emissions, and NYC-specific concentration–response (C-R) functions for asthma ED visits^50,51^ and cardiovascular hospital admissions.^23,51^ ZAPPA estimates change in air pollution-related health impacts and the economic value of these impacts using an approach that is consistent with EPA regulatory impact analyses.^45,52^ Incidence data for HIA consisted of nine different end points: adult and infant mortality; nonfatal heart attacks; respiratory-related and cardiovascular-related hospitalizations; acute bronchitis; upper and lower respiratory symptoms; asthma-related ED visits; asthma exacerbations; minor restricted activity days (i.e., days on which activity is reduced, but not severely restricted); and workdays lost due to illness (Table S9). Additional details on these functions can be found in the COBRA manual.^43^ We used 3-year annual average rates (2015–2017) at the ZCTA-level for the following outcomes: rate of asthma ED visits, all-cause mortality, and respiratory and cardiovascular hospitalizations. The remaining baseline rates are at the county level (2016) from the COBRA tool (see Table S8). All benefits are annual counts based on current population and incidence with no projected data.

## Results

### 2016 Baseline

**Figure 3:**
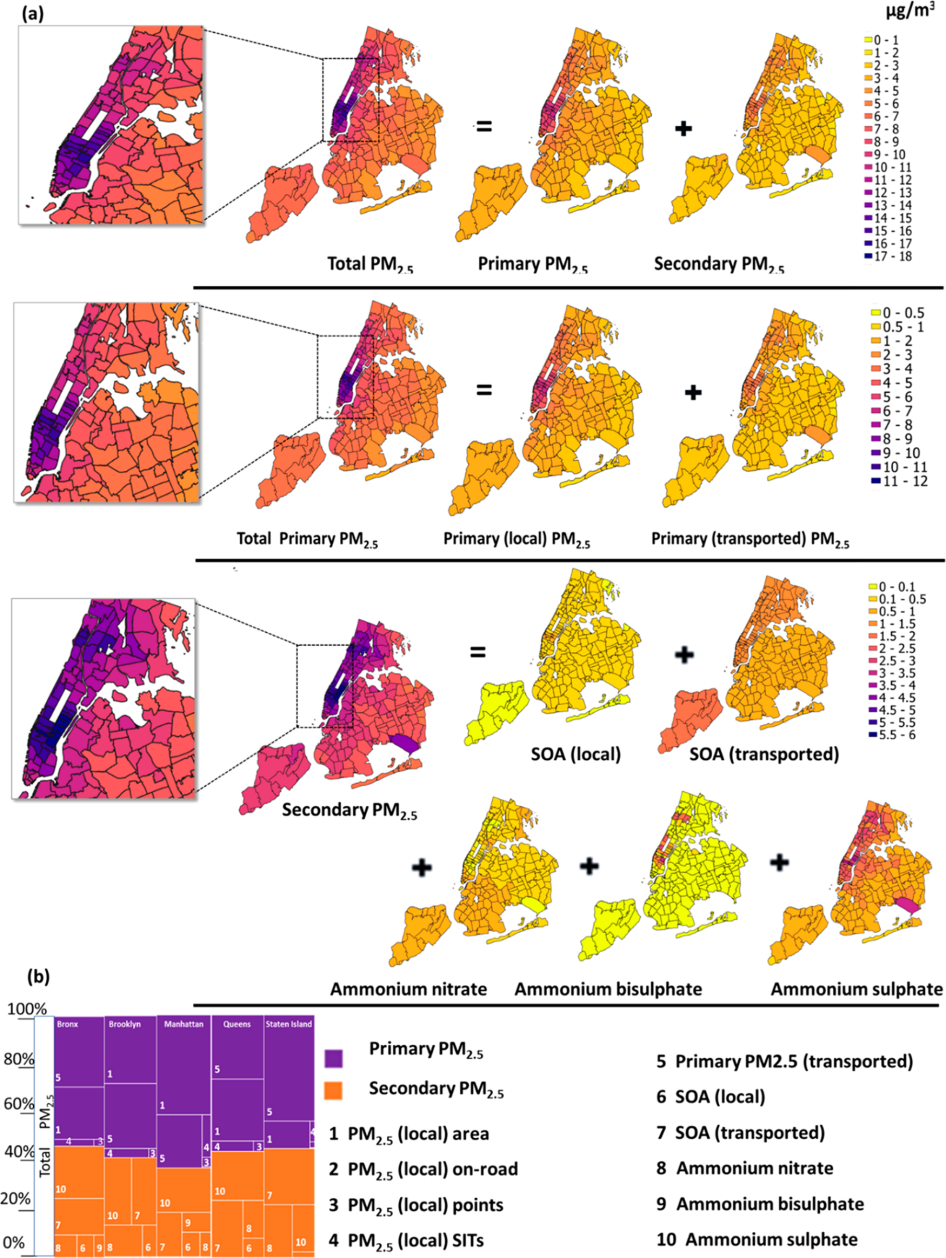
(a) Baseline (2016) ZCTA-level total PM_2.5_: Primary and secondary PM_2.5_ components and (b) borough-level aggregated overview of primary and secondary PM_2.5_ components.^45^

Based on ZAPPA, NYC experiences an annual average of 8.0 ± 3.3 μg/m^3^ total PM_2.5_ across ZCTAs. Primary (direct) emissions make up the majority (59%) of citywide PM_2.5_, which includes contribution from both local (35% of citywide PM_2.5_) and transported sources (24% of citywide PM_2.5_) (Figure 3). The local sources of primary PM_2.5_ include area sources (90%, mostly building emissions), on-road sources (8%), electrical generating units (EGU) emissions (1%), and ships in transit (<1%). The local (in situ) generation of primary PM_2.5_ is highest in the ZCTAs of Bronx and Manhattan (average ZCTA concentration in Bronx, 1.9 ± 0.6 μg/m^3^, and Manhattan, 5.7 ± 1.8 μg/m^3^). Secondary PM_2.5_ (Figure 3a and 3b) comprises 41% of the citywide total. Of the secondary PM_2.5_ species, the contribution from ammonium sulfate (45%) is highest, followed by secondary organic aerosols (SOA) (40%) and ammonium nitrate (15%). The ZCTAs of Manhattan and Staten Island are the most influenced by transport, evidenced by the higher concentrations of transported primary PM_2.5_ and transported SOA in these boroughs (Figure 3a and 3b). This is due to the influence of westerly winds and increased turbulence attributed to complex geography aided by water bodies and high-rise buildings. Figure 3 shows that the transported components of PM_2.5_ decrease moving eastward.

The quantitative evaluation (Table 1) of total PM_2.5_ concentrations demonstrates that the modeled baseline is well within desired acceptance criteria of previous studies when compared against monitored concentrations (EPA and NYCCAS), thus showing the robustness of our modeled estimates.

### Results from Five Policy-Based Emissions Reduction Scenarios

#### Sc_1_ Commercial Charbroiling

NYC-based studies^13,25^ identified commercial cooking as a major source of PM_2.5_ throughout the city. Commercial cooking (4204 tons per year (tpy)) emissions contribute about 37% of locally emitted PM_2.5_,^53^ the single highest contribution from any SCC category in NYC.^54^ In 2015, NYC took a step forward in addressing this pollutant source when New York City Council passed Local Law 38 of 2015.^55^ The law updated the NYC Air Code to include regulation of commercial cooking emissions, requiring the installation of emission control devices on grills and char broilers in restaurants cooking significant amounts of meat with an expected reduction of 75% PM_2.5_ emissions.

**Figure 4:**
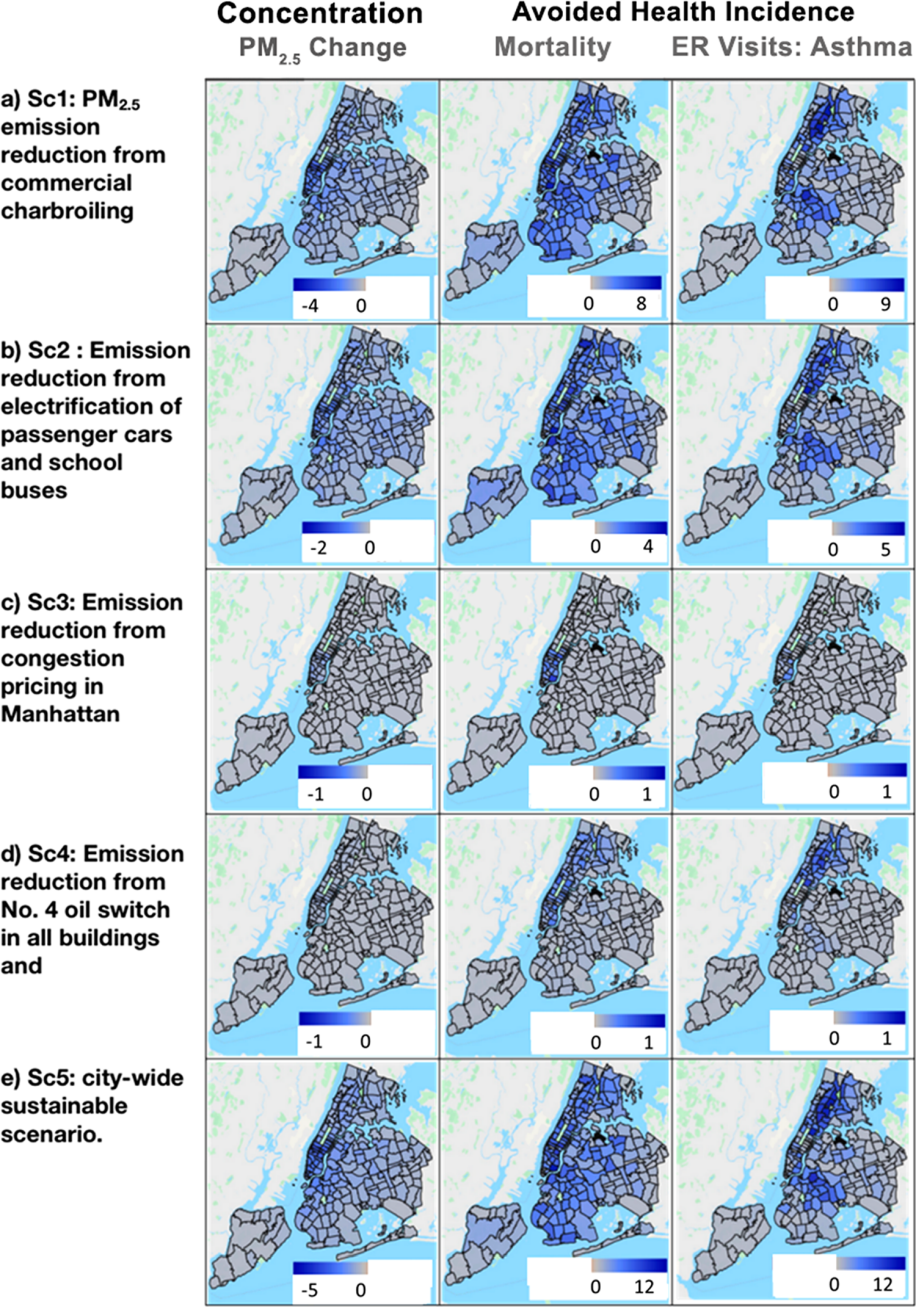
Avoided health incidences (mortality and ER visits due to asthma) because of the PM_2.5_ change (μg/m^3^) after implementing strategy-based scenarios in NYC using ZAPPA

We applied ZAPPA to the scenario of full implementation of this law and found a reduction of 7.6%, (0.6 ± 0.5 μg/m^3^) in average PM_2.5_ across all 192 ZCTAs (Table 2). The widespread reduction reflects the presence of commercial charbroiling in all five counties (Figure 4a), with the largest reductions in neighborhoods with the most restaurants. Drilling down geographically, specific ZCTAs (Figure S4) in Manhattan (ID 10165, 10170, 10020, and 10110) and Brooklyn (Kings County) (ID 11217, 11201, and 11211) were identified as accruing significantly greater reductions than average (14–37% of base PM_2.5_). This scenario had the greatest potential health benefits, including the greatest number of avoided mortalities per year (144–324 deaths) (Table 2) and an estimated an annual monetary benefit of $3.5B, with $3.4B from avoided mortality (Table S10). ZAPPA results (Figures 4a and S5a) show the variable distribution of these benefits within counties, pointing to the tool’s ability to support future analyses of inequitable air pollution exposures and impacts in NYC, as has been documented in other studies.^56,57^

**Table 2:** Scenario-Based Declines in Annual Average Ambient PM_2.5_ Levels for NYC, Associated Avoided Health Outcomes and Monetized Benefits Per Year

avoided event/scenario	Sc1 emission reduction from charbroiling	Sc2 emission reduction from electrification of passenger cars and school buses	Sc3 emission reduction from congestion pricing in Manhattan	Sc4 emission reduction from no. 4 oil switch in all buildings	Sc5 city-wide sustainable scenario Sc1 + Sc2 + Sc3 + Sc4
city average delta (μ ± σ) PM_2.5_min–max ZCTA μg/m^3^)	0.6 ± 0.5	0.3 ± 0.1	0.01 ± 0.04	0.03 ± 0.02	0.9 ± 0.7
	0.06–3.52	0.01–1.09	0.00–0.18	0.00–0.10	0.08–4.14
mortality (low estimate)	143	74	3	7	210
mortality (high estimate)	324	168	6	15	475
infant mortality	1	0	1	0	1
nonfatal heart attacks (low estimate)	15	8	3	1	22
nonfatal heart attacks (high estimate)	137	72	1	6	200
hospital admits, all respiratory	37	19	1	2	55
hospital admits, all respiratory direct	15	8	1	1	22
hospital admits, asthma	10	5	1	1	15
hospital admits, chronic lung disease	13	6	1	1	19
hospital admits, cardio (except heart attacks)	33	18	1	1	49
acute bronchitis	235	123	3	10	341
upper respiratory symptoms	4271	2224	44	180	6,206
lower respiratory symptoms	3003	1569	31	127	4,355
emergency room visits, asthma	235	120	3	13	343
minor restricted activity days	179084	88847	3527	7720	259212
work loss days	30950	15292	616	1330	44784
asthma exacerbation	4419	2296	47	188	6420
asthma exacerbation, cough	1003	521	11	43	1458
asthma exacerbation, shortness of breath	1351	702	15	57	1963
asthma exacerbation, wheeze	2065	1073	22	88	3000
overall health benefits (U.S dollars $)	$3.5 B	$1.8 B	$62 M	$0.15 B	$5.1 B

#### Sc_2_ Electrification of Passenger Vehicles

On-road mobile sources account for more than 10–16% of total PM_2.5_ mass concentrations (and about ∼52% of EC and ∼25% of NOx emissions) in NYC.^30^ The NEI 2016 EPA inventory shows that approximately 87% of passenger cars are fueled by gasoline. This scenario assesses the potential effects of 100% electrification of private passenger cars and school buses in NYC. We found that the total PM_2.5_ citywide average would fall by 0.3 ± 0.1 μg/m^3^ (highest decrease, 1.0 μg/m^3^ in ID 11427; lowest, 0.01 in 10308) (Figure 4b). The spatial pattern of the reductions generally followed traffic density with the greatest decreases in central Queens (ID 114271.0 μg/m^3^), Manhattan (ID 10022–0.9 μg/m^3^), and Brooklyn (ID 11201–0.8 μg/m^3^), all locations where major roads or bridge approaches intersect. Health benefits follow a similar pattern to the PM_2.5_ reductions with avoided premature deaths well distributed across most of the city. The greatest number of avoided ED visits for asthma were seen in the ZCTAs of northern Manhattan and southern Bronx where baseline rates tend to be highest. We estimated a total monetary benefit of $1.8B, with $1.78 B per year from mortality alone (Table S10 and Figure S5b).

#### Sc_3_ Congestion Pricing in Midtown Manhattan

The Central Business District Tolling Program (CBDTP), the nation’s first congestion pricing program, will start in NYC in 2022 and will require vehicles to pay to enter midtown and downtown Manhattan, potentially reducing congestion and improving air quality. The scenario we tested reduces the traffic volume AADT (annual average daily traffic) by 15% (based on public estimates^58^) in the 30 ZCTAs in the zone–an analysis comprising only part of a county for air pollution and health benefit estimation. PM_2.5_ reduction across the ZCTAs ranges between 0.00 and 0.18 μg/m^3^ (Table 2 and Figure 4c). Highest PM_2.5_ reduction (an average of 0.15 μg/m^3^) was observed in the ZCTAs of lower Manhattan (ID 10111, 10165, and 10170, Figure S4). Overall, this scenario resulted in six fewer mortalities and three fewer asthma ED visits in the midtown Manhattan per year (Table 2 and Figure 4c). This scenario illustrates the contribution that ZAPPA can make to cost-benefit analyses for a policy yielding two kinds of monetized benefits: indirect health benefits attributable to air pollution reductions (Table S10 and Figure S5c) and direct overall monetized benefits (proposed to be about $15B) due in this case to increased toll pricing.^58^ However, this does not account for the anticipated health benefits from the improved public transit system that should result from increased funding.

#### Sc_4_ No. 4 Oil Prohibition in All Commercial and Residential Buildings

This scenario involves the prohibition of all No. 4 oil (a blend of residual and distillate fuel oils) in NYC buildings for heating and hot water production as required by Local Law 43 of 2010.^58^ We calculated the citywide PM_2.5_ emissions coming solely from No. 4 oil for each borough: Manhattan (45.4 tpy), Bronx (23.2 tpy), Queens (11.9 tpy), Brooklyn (7.3 tpy), and Staten Island (1.4 tpy), and reduced them to zero. We found an average reduction in average PM_2.5_ of 0.01–0.10 μg/m^3^ (Table 2 and Figure 4d) across all 192 ZCTAs with substantial variation within counties. This scenario resulted in spatially nonhomogeneous reduction due to high-rise buildings dominating the ZCTAs of lower Manhattan (highest in ID 10110 and 10036: 0.1 μg/m^3^) and Bronx (highest in ID 10453 and 10452:0.05 μg/m^3^). The scenario avoided a total 15 deaths and asthma ED visits per year (Table 2). We estimated a total annual monetary benefit of $156 M (higher estimate), with $153 M only from mortality (Table S11 and Figure S5d).

#### Sc_5_ City-Wide Sustainable Scenario

The fifth (Sc_5_) policy-based scenario involved the combined implementation of the four previous scenarios (commercial cooking emissions controls, electrification of passenger vehicles, congestion pricing in midtown and lower Manhattan, and No. 4 oil prohibition in all buildings). This scenario would reduce PM2.5 emissions by 1930 tpy citywide and would result in a citywide average decrease in ambient PM2.5 of 11%, that is, 0.9 μg/m^3^ (Table 2). The highest reduction per ZCTA (4.12 μg/m^3^) was in ZCTA ID 10165 (lower Manhattan) (Figure 4e), while the lowest was in 10307 (0.08 μg/m^3^) in southern Staten Island. A cluster of ZCTAs in mid and lower Manhattan (10075, 10110, 10111, 10128, 10162, 10165, 10170, 10171, 10199, 10278, and 10280) had an average reduction of 2.3 μg/m^3^ because of the concentration of targeted emissions sources in a region of high population density. ZCTAs in central Brooklyn (ZCTA ID 11201, 11217, 11205, and surrounding), northeast Queens (ZCTA ID 11103, 11424, and 11104), and southern Bronx (ZCTA ID 10458 and 10451) also had large reductions in health risks like mortality (ZCTA ID 10002, 10016, and 10022 in Manhattan avoided about 10 deaths each) and asthma ED visits (ZCTA ID 10457 and 10456 in the Bronx avoid about 11 asthma ED visits). Although the reduction in PM_2.5_ concentrations in some ZCTAs (especially central and southern Brooklyn) was smaller when compared to reductions in lower Manhattan, end points like asthma ED visits and respiratory admissions showed larger reductions because of higher baseline rates of those health outcomes. This scenario estimates an average reduction in PM_2.5_ of 0.9 μg/m^3^, which translates to avoiding 210–475 (upper–lower bound) deaths and 340 asthma emergency department visits. The monetized health benefits range from $2B–5B annually.

## Discussion

We developed ZAPPA, a novel high-resolution modeling framework that combines the two reduced-form models C-TOOLS and COBRA, to facilitate rapid policy assessment at the neighborhood, as opposed to county, level. We used NYC-specific emission inventories, city population demographics and health incidence data to ensure the most specific achievable estimates for small-areas. ZAPPA estimates ZCTA-level total PM_2.5_ in NYC, while accounting for primary and secondary PM_2.5_ from local and transported components into the city. Modeled PM_2.5_ compared well against routine monitoring data, using multiple measures of model performance.

We used ZAPPA to test the impacts of various illustrative emission-reduction strategies based on existing sector-specific policies for buildings, on-road, and commercial cooking sources.^23^ We demonstrated how ZAPPA can be used to compare estimated health savings from proposed policies, and support emissions-based sensitivity analyses for the development of new policies (Figure S6). The spatial variability of benefits across ZCTAs is driven by baseline concentrations from all emission sources, design of the emissions reduction policy/program, and variation in baseline rates of health outcomes. When all four scenarios were combined (Sc_5_), median reduction in ambient PM2.5 at the ZCTA level ranged from 0.08 (ID 10307 in Staten Island) to 4.1 μg/m^3^ (ID 10165 in Manhattan). Health benefits were similarly variable, with many avoided ED visits and hospitalizations occurring in Brooklyn and Bronx ZCTAs where baseline health incidence rates are high. The maximum monetized benefits ranged from $0.01 M (ID 11430 Queens) to $56 M (ID 10002 Manhattan). Regulation of commercial cooking emissions from commercial charbroiling (Sc1) had the largest PM_2.5_ reductions of the sector-specific scenarios, ranging from 0.06 μg/m^3^ (ID 11307 in Richmond and 11697 in Queens) to 3.52 μg/m^3^ (ID 10165). It has been reported^59^ that restaurants cause long-term average PM_2.5_ increases of 0.1 to 0.3 μg/m^3^ over distances between 50 and 450 m, and ZAPPA allows users to estimate accrued health benefits to the local neighborhoods of emissions reduction measures.

In on-road scenarios Sc2, 100% electrification of cars and school buses, and Sc3, 15% AADT reduction Central Business District due to congestion pricing, we saw a wide range of reductions (highest 1.09 μg/m^3^ (ZCTA ID 11427 Queens) and lowest 0.01 μg/m^3^ (ZCTA ID 10308 Staten Island)) across neighborhoods. The PM_2.5_ reductions from Sc2, a fleet-wide emissions reduction scenario, were widely distributed across ZCTAs, as were the health benefits. In comparison, the PM_2.5_ reductions and associated health benefits from Sc3 were limited to ZCTAs in lower and midtown Manhattan, where traffic volume was reduced by congestion pricing (Figure 4c). Like the scenarios illustrated here, the Transportation Climate Initiative (TCI^60^) has modeled regional policy scenarios at state and county scales, showing reductions in mortality with implementation of cap-and-invest programs. ZAPPA’s ZCTA-level analysis could be used to better estimate the health impact of these policy scenarios at a neighborhood level and for populations with disproportionate exposure to air pollution. Given evidence of disproportionate health outcomes in low-income communities in NYC,^30^ scenarios Sc2 and Sc3 suggest that policy solutions are most effective when they impact traffic in neighborhoods with the highest baseline rates of air pollution-related health outcomes, such as the electrification of medium-duty and heavy-duty trucks or low and zero emissions zones in heavily residential neighborhoods.

Our fourth scenario to prohibit of No. 4 oil in all buildings (Sc4) illustrates the extension of existing building fuel policies and evaluation of potential benefits. This strategy’s analysis showed widespread health benefits due to the spatial correlation between population and building density in NYC. However, we found significantly greater impacts in the ZCTAs of northern Manhattan and the south Bronx where the majority of buildings burning No. 4 oil are located. The stack height of building boilers is closer to pedestrian level than point-based emission sources (i.e., power plants), making buildings emissions especially relevant to NYC public health. While the overall PM_2.5_ reduction is less significant and nonuniform in this scenario, it is one of the most viable scenarios^61^ for NYC regulation from an economic perspective, since cleaner fuel alternatives are available and competitively priced. While the total ambient PM_2._ concentration in NYC is a function of both local/in situ and transported emissions, all emissions reductions scenarios presented here only target local emissions, limited to the reach of NYC policy impacts. Overall, about 11% reduction in total PM_2.5_ (0.9 μg/m^3^) is estimated when all four scenarios are implemented together. This reduction translates into achieving $2B–5B monetized health benefits annually.

ZAPPA shows efficient integration and usage of local data sets in studying NYC’s air quality and health burden; however, we acknowledge that there are uncertainties associated with C-R functions, baseline health rates, NEI-based emissions inventory, and in our methodical approach to spatially distribute emissions to ZCTA that ZAPPA does not quantify. While we have shown the impact of illustrative emissions scenarios, they do not take system-wide changes into account. For exmple, electrification of cars and buses shows direct benefits from this scenario, while there are potential offsets due to increase in emissions in the power sector depending on the fuel used. These limitations can be addressed in future work by coupling with energy systems modeling or other approaches. The innovation of ZAPPA is that it combines two robust reduced-form models and is designed for rapid assessments of a diverse range of emissions reduction scenarios to inform policy development. While ZAPPA makes a significant advance in estimating concentrations of primary pollutants at high resolution from local sources, as in other reduced form models,^62^ it has limitations with estimating secondary aerosols due to various simplifying assumptions about the emissions to air quality relationships. For this study, we have used VOC to SOA emissions conversion and then dispersed the converted SOA emissions to estimate SOA concentrations. This approach is comparable to other reduced form models in the literature.^56,63^ Additionally, while C-TOOLS-based dispersion estimates for local NYC sources are at ZCTA level, the transported components from outside the city are county-level estimates from COBRA, which we believe is a reasonable approximation. Despite these limitations, the significant advantage that ZAPPA has in allowing the user to rapidly evaluate multiple policy options for screening purposes is to be highlighted.

We used ZAPPA to test a suite of scenarios based on existing air quality and sustainability policies to demonstrate its effectiveness as a screening tool. Beyond quantified health benefit estimates, ZAPPA also provides estimates of cost savings from monetized health benefits. ZAPPA has several unique benefits compared to typical chemical transport models, such as resolution (ZCTA-level emissions and population embedded results), efficiency (faster run time), ease of use (web-based scenario development), and integrated assessment (integrating air quality and health benefits modeling in a single framework), making it a powerful tool for comparing policy options and ensuring that benefits are distributed equitably.^64,65^ ZAPPA’s ability to model health benefits at ZCTA-level offers a strong potential for studying environmental justice issues, given previous findings that both the population-weighted average exposure and the exposure differences between minorities and whites increased substantially when switching from the coarsest to the finest resolution grid.^66^

We anticipate that ZAPPA can be expanded to other cities in the U.S. and the world for assessing PM_2.5_-based health impacts, designing emissions reduction scenarios, and to further identify vulnerable populations with disproportionate exposures to air pollution sources. Specifically, future extensions of ZAPPA can add emerging knowledge on new adverse health impacts of exposure to PM_2.5_ and incorporate treatment for additional pollutants, such as NO_2_ from combustion sources, which are also known to cause adverse human health impacts.^67^

## Supporting Information Available

Additional tables and figures on emissions inputs, health data and health impact studies, and results from illustrative scenarios (PDF)

## References

[ref1] Xiao Q.; Chen H.; Strickland M. J.; Kan H.; Chang H. H.; Klein M.; Yang C.; Meng X.; Liu Y. Associations between birth outcomes and maternal PM2. 5 exposure in Shanghai: A comparison of three exposure assessment approaches. Environment International 2018, 117, 226–236. 10.1016/j.envint.2018.04.050. 29763818 PMC6091210

[ref2] Cesaroni G.; Forastiere F.; Stafoggia M.; Andersen Z. J.; Badaloni C.; Beelen R.; Caracciolo B.; de Faire U.; Erbel R.; Eriksen K. T.; Fratiglioni L.; Galassi C.; Hampel R.; Heier M.; Hennig F.; Hilding A.; Hoffmann B.; Houthuijs D.; Jockel K.-H.; Korek M.; Lanki T.; Leander K.; Magnusson P. K. E.; Migliore E.; Ostenson C.-G.; Overvad K.; Pedersen N. L.; J J. P.; Penell J.; Pershagen G.; Pyko A.; Raaschou-Nielsen O.; Ranzi A.; Ricceri F.; Sacerdote C.; Salomaa V.; Swart W.; Turunen A. W.; Vineis P.; Weinmayr G.; Wolf K.; de Hoogh K.; Hoek G.; Brunekreef B.; Peters A. Long term exposure to ambient air pollution and incidence of acute coronary events: prospective cohort study and meta-analysis in 11 European cohorts from the ESCAPE Project. BMJ 2013, 348, f7412 10.1136/bmj.f7412. PMC3898420 24452269

[ref3] Gardner B.; Ling F.; Hopke P. K. Ambient fine particulate air pollution triggers ST-elevation myocardial infarction, but not non-ST elevation myocardial infarction: A case-crossover study. Particle and Fibre Toxicology 2014, 10.1186/1743-8977-11-1. PMC3891992 24382024

[ref4] Lepeule J.; Laden F.; Dockery D.; Schwartz J. Chronic exposure to fine particles and mortality: An extended follow-up of the Harvard six cities study from 1974 to 2009. Environmental Health Perspectives. 2012, 120 (7), 965–970. 10.1289/ehp.1104660. 22456598 PMC3404667

[ref5] Thurston G. D.; Burnett R. T.; Turner M. C.; Shi Y.; Krewski D.; Lall R.; Ito K.; Jerrett M.; Gapstur S. M.; Diver W. R.; Pope C. A. Ischemic heart disease mortality and long-term exposure to source-related components of US fine particle air pollution. Environmental Health Perspectives 2016, 124 (6), 785–794. 10.1289/ehp.1509777. 26629599 PMC4892920

[ref6] Vohra K.; Vodonos A.; Schwartz J.; Marais E. A.; Sulprizio M. P.; Mickley L. J. Global mortality from outdoor fine particle pollution generated by fossil fuel combustion: Results from GEOS-Chem. Environmental Research. 2021, 195, 110754 10.1016/j.envres.2021.110754. 33577774

[ref7] Dedoussi I. C.; Eastham S. D.; Monier E.; Barrett S. R. H. Premature mortality related to United States cross-state air pollution. Nature 2020 578:7794. 2020, 578 (7794), 261–265. 10.1038/s41586-020-1983-8. 32051602

[ref8] Cohen A.; Brauer M.; Burnett R.; Lancet H. A. T. Estimates and 25-year trends of the global burden of disease attributable to ambient air pollution: an analysis of data from the Global Burden of Diseases. Lancet 2017, 389 (10082), 1907–1918. 10.1016/S0140-6736(17)30505-6. 28408086 PMC5439030

[ref9] Fine P. M.; Sioutas C.; Solomon P. A. Secondary Particulate Matter in the United States: Insights from the Particulate Matter Supersites Program and Related Studies Secondary Particulate Matter in the United States: Insights from the Particulate Matter Supersites Program and Related Studies. Journal of the Air & Waste Management Association 2008, 58, 234–253. 10.3155/1047-3289.58.2.234. 18318339

[ref10] Bell M. L.; Dominici F.; Ebisu K.; Zeger S. L.; Samet J. M. Spatial and temporal variation in PM2.5 chemical composition in the United States for health effects studies. Environmental Health Perspectives. 2007, 115 (7), 989–995. 10.1289/ehp.9621. 17637911 PMC1913582

[ref11] Arter C. A; Buonocore J.; Chang C.; Arunachalam S. Mortality-based damages per ton due to the on-road mobile sector in the Northeastern and Mid-Atlantic US by region, vehicle class and precursor. Environ. Res. Lett. 2021, 16, 065008 10.1088/1748-9326/abf60b.

[ref12] Blanchard C. L.; Shaw S. L.; Edgerton E. S.; Schwab J. J. Ambient PM2.5 organic and elemental carbon in New York City: changing source contributions during a decade of large emission reductions. J. Air Waste Manage. Assoc. 2021, 71, 995 10.1080/10962247.2021.1914773. 33835900

[ref13] Pitiranggon M.; Johnson S.; Haney J.; Eisl H.; Ito K. Long-term trends in local and transported PM2.5 pollution in New York City. Atmos. Environ. 2021, 248, 118238 10.1016/j.atmosenv.2021.118238.

[ref14] Ito K.; Johnson S.; Kheirbek I.; Clougherty J.; Pezeshki G.; Ross Z.; Eisl H.; Matte T. D. Intraurban variation of fine particle elemental concentrations in New York City. Environ. Sci. Technol. 2016, 50 (14), 7517–7526. 10.1021/acs.est.6b00599. 27331241

[ref15] Rattigan O. V.; Civerolo K. L.; Felton H. D.; Schwab J. J.; Demerjian K. L. Long term trends in New York: PM2. 5 mass and particle components. Aerosol Air Qual. Res. 2016, 16, 1191–1205. 10.4209/aaqr.2015.05.0319.

[ref16] Squizzato S.; Masiol M.; Rich D.; Environment P. H. A. A long-term source apportionment of PM2. 5 in New York State during 2005–2016. Atmospheric Environment 2021, 192, 35–47. 10.1016/j.atmosenv.2018.08.044.

[ref17] Masiol M.; Squizzato S.; Rich D. Q.; Hopke P. K. Long-term trends (2005–2016) of source apportioned PM2.5 across New York State. Atmos. Environ. 2019, 201, 110–120. 10.1016/j.atmosenv.2018.12.038.

[ref18] Adhikari A.; Yin J. Short-Term Effects of Ambient Ozone, PM2.5, and Meteorological Factors on COVID-19 Confirmed Cases and Deaths in Queens, New York. International Journal of Environmental Research and Public Health 2020, Vol 17, Page 4047 2020, 17 (11), 4047 10.3390/ijerph17114047. PMC7312351 32517125

[ref19] Hopke P. K.; Dai Q.; Li L.; Feng Y. Global review of recent source apportionments for airborne particulate matter. Science of The Total Environment. 2020, 740, 140091 10.1016/j.scitotenv.2020.140091. 32559544 PMC7456793

[ref20] Hopke P.; Croft D.; Zhang W.; Lin S. Changes in the acute response of respiratory diseases to PM2. 5 in New York State from 2005 to 2016. Science of the Total Environment 2021, 677, 328–339. 10.1016/j.scitotenv.2019.04.357. 31059876

[ref21] Kheirbek I.; Haney J.; Douglas S.; Ito K.; Caputo S.; Matte T. The public health benefits of reducing fine particulate matter through conversion to cleaner heating fuels in New York City. Environ. Sci. Technol. 2014, 48 (23), 13573–13582. 10.1021/es503587p. 25365783

[ref22] Weber S. A.; Insaf T. Z.; Hall E. S.; Talbot T. O.; Huff A. K. Assessing the impact of fine particulate matter (PM2.5) on respiratory-cardiovascular chronic diseases in the New York City Metropolitan area using Hierarchical Bayesian Model estimates. Environmental Research 2016, 151, 399–409. 10.1016/j.envres.2016.07.012. 27543787

[ref23] Ito K.; Mathes R.; Ross Z.; Nádas A.; Thurston G.; Matte T. Fine particulate matter constituents associated with cardiovascular hospitalizations and mortality in New York City. Environmental Health Perspectives. 2011, 119 (4), 467–473. 10.1289/ehp.1002667. 21463978 PMC3080927

[ref24] Huang K.; Bi J.; Meng X.; et al. Estimating daily PM2.5 concentrations in New York City at the neighborhood-scale: Implications for integrating non-regulatory measurements. Science of The Total Environment. 2019, 697, 134094 10.1016/j.scitotenv.2019.134094. 32380602

[ref25] Johnson S.; Haney J.; Cairone L.; Huskey C.; Kheirbek I. Assessing Air Quality and Public Health Benefits of New York City’s Climate Action Plans. Environmental Science & Technology. 2020, 54 (16), 9804–9813. 10.1021/acs.est.0c00694. 32663397

[ref26] Jin X.; Fiore A. M.; Civerolo K.; et al. Comparison of multiple PM2.5 exposure products for estimating health benefits of emission controls over New York State, USA. Environmental Research Letters. 2019, 14 (8), 084023 10.1088/1748-9326/ab2dcb.

[ref27] Kheirbek I. The New York City Community Air Survey. Google Scholar. https://scholar.google.com/scholar_lookup?title=The%20New%20York%20city%20community%20air%20Survey%3A%20neighborhood%20air%20quality%202008-2017&publication_year=2019&author=NYCDOHMH%20(New%20York%20City%20Department%20of%20Health%20and%20Mental%20Hygiene) (accessed 2021-07-13).

[ref28] NYC Department of Health and Mental Hygiene Data Portal. https://a816-dohbesp.nyc.gov/IndicatorPublic/Subtopic.aspx?theme_code=23&subtopic_id=103.

[ref29] Jin X.; Fiore A. M.; Civerolo K.; et al. Comparison of multiple PM2.5 exposure products for estimating health benefits of emission controls over New York State, USA. Environmental Research Letters 2019, 14, 084023 10.1088/1748-9326/ab2dcb.

[ref30] Kheirbek I.; Haney J.; Douglas S.; Ito K.; Matte T. The contribution of motor vehicle emissions to ambient fine particulate matter public health impacts in New York City: A health burden assessment. Environmental Health: A Global Access Science Source 2016, 10.1186/s12940-016-0172-6. PMC5002106 27566439

[ref31] Zhao C.; Wang Q.; Ban J.; et al. Estimating the daily PM2.5 concentration in the Beijing-Tianjin-Hebei region using a random forest model with a 0.01° × 0.01° spatial resolution. Environment International. 2020, 134, 105297 10.1016/j.envint.2019.105297. 31785527

[ref32] Arter C. A.; Arunachalam S. Assessing the importance of nonlinearity for aircraft emissions’ impact on O3 and PM2.5. Science of The Total Environment. 2021, 777, 146121 10.1016/j.scitotenv.2021.146121.

[ref33] Doraiswamy P.; Hogrefe C.; Hao W.; Civerolo K.; Ku J. Y.; Sistla G. A Retrospective Comparison of Model-Based Forecasted PM2.5 Concentrations with Measurements. Journal of the Air & Waste Management Association 2010, 60 (11), 1293–1308. 10.3155/1047-3289.60.11.1293. 21141423

[ref34] Lightstone S.; Gross B.; Moshary F.; Castillo P. Development and Assessment of Spatially Continuous Predictive Algorithms for Fine Particulate Matter in New York State. Atmosphere 2021, Vol 12, Page 315 2021, 12 (3), 315 10.3390/atmos12030315.

[ref35] Weber S. A.; Insaf T. Z.; Hall E. S.; Talbot T. O.; Huff A. K. Assessing the impact of fine particulate matter (PM2.5) on respiratory-cardiovascular chronic diseases in the New York City Metropolitan area using Hierarchical Bayesian Model estimates. Environmental Research. 2016, 151, 399–409. 10.1016/j.envres.2016.07.012. 27543787

[ref36] Chang S.; Vizuete W.; Valencia A.; et al. A modeling framework for characterizing near-road air pollutant concentration at community scales. Science of the Total Environment 2015, 538, 905–921. 10.1016/j.scitotenv.2015.06.139. 26363146

[ref37] Clougherty J.; Kheirbek I.; Eisl H.; et al Intra-urban spatial variability in wintertime street-level concentrations of multiple combustion-related air pollutants: the New York City Community Air Survey. Journal of Exposure Science & Environmental Epidemiology 2013, 23, 232–240. 10.1038/jes.2012.125. 23361442

[ref38] Huang K.; Bi J.; Meng X.; et al. Estimating daily PM2.5 concentrations in New York City at the neighborhood-scale: Implications for integrating non-regulatory measurements. Science of The Total Environment 2019, 697, 134094 10.1016/j.scitotenv.2019.134094. 32380602

[ref39] Barzyk T. M.; Isakov V.; Arunachalam S.; Venkatram A.; Cook R.; Naess B. A Near-Road Modeling System for Community-Scale Assessments of Mobile-Source Air Toxics: The Community Line Source (C-LINE) Modeling System. Environ. Model. Software 2015, 66, 46–56. 10.1016/j.envsoft.2014.12.004.

[ref40] Isakov V.; Barzyk T. M.; Smith E. R.; Arunachalam S.; Naess B.; Venkatram A. A web-based screening tool for near-port air quality assessments. Environ. Model. Software 2017, 98, 21 10.1016/j.envsoft.2017.09.004. PMC5906819 29681760

[ref41] Sorte S.; Arunachalam S.; Naess B.; et al. Assessment of source contribution to air quality in an urban area close to a harbor: Case-study in Porto, Portugal. Science of The Total Environment. 2019, 662, 347–360. 10.1016/j.scitotenv.2019.01.185. 30690369

[ref42] U.S. EPA. Technical Support Document (TSD) Preparation of Emissions Inventories for 2016v1 North American Emissions Modeling Platform Technical Report, 2020. www.epa.gov/sites/production/files/2020-10/documents/2016v1_emismod_tsd_508.pdf (accessed 2021-07-13).

[ref43] U.S. EPA. User’s Manual for the Co-benefits Risk Assessment (COBRA) Screening Model Version: 4.1, 2021. https://www.epa.gov/system/files/documents/2021-11/cobra-user-manual-nov-2021_4.1_0.pdf.

[ref44] U.S. EPA NEI. Emissions inventories for 2016v1 North American emissions modeling platform. https://www.epa.gov/sites/production/files/2020-11/documents/2016v1_emismod_tsd_508.pdf.

[ref45] U.S. EPA. U.S. EPA-default options for PM2.5 impact assessments: BenMap configuration (.cfg) file; Office of Air Quality Planning and Standards, Risk and Benefits Group: Research Triangle Park, NC. https://www.epa.gov/sites/default/files/2014-12/pm25o3no2so2configurations.zip.

[ref46] Baek B. H.; Seppanen C. Sparse Matrix Operator Kernel Emissions (SMOKE) Modeling System. Zenodo 2018, 10.5281/ZENODO.1421403.

[ref47] U.S. EPA. User’s Guide for the AERMOD Meteorological Preprocessor (AERMET), 2021. https://gaftp.epa.gov/Air/aqmg/SCRAM/models/met/aermet/aermet_userguide.pdf.

[ref48] Childs P. P.; Raman S. Observations and Numerical Simulations of Urban Heat Island and Sea Breeze Circulations over New York City. pure and applied geophysics 2005 162:10. 2005, 162 (10), 1955–1980. 10.1007/s00024-005-2700-0.

[ref49] Chang J. C.; Hanna S. R. Air quality model performance evaluation. Meteorology and Atmospheric Physics 2004 87:1. 2004, 87 (1), 167–196. 10.1007/s00703-003-0070-7.

[ref50] Ito K.; et al. Characterization of PM 2.5, gaseous pollutants, and meteorological interactions in the context of time-series health effects models. Journal of Exposure Science & Environmental Epidemiology 2021, 17, S45–S60. 10.1038/sj.jes.7500627. 18079764

[ref51] NYCDOHMH (New York City Department of Health and Mental Hygiene. The New York city community air Survey: neighborhood air quality 2008–2017. https://nyc-ehs.net/besp-report/web/nyccas.

[ref52] U.S. EPA. U.S. EPA-default options for PM2.5 impact assessments: BenMap aggregation, pooling and valuation (.apv) file; Office of Air Quality Planning and Standards, Risk and Benefits Group: Research Triangle Park, NC. https://www.epa.gov/sites/default/files/2014-12/pm25o3no2so2configurations.zip.

[ref53] National Emission Inventory, 2016. https://www.epa.gov/air-emissions-inventories/2017-national-emissions-inventory-nei-data.

[ref54] NYCDOHMH. The New York City Community Air Survey: Neighborhood Air Quality 2008–2017. https://nyc-ehs.net/besp-report/web/nyccas.

[ref55] Manuel A.; Santiago R. New DEP Rule Targets Exhaust Emissions from Commercial Kitchens. https://www.milrose.com/insights/new-dep-rule-targets-exhaust-emissions-from-commercial-kitchens.

[ref56] Tessum C. W.; Hill J. D.; Marshall J. D. InMAP: A model for air pollution interventions. PLoS One 2017, 12 (4), e0176131 10.1371/journal.pone.0176131. 28423049 PMC5397056

[ref57] Colmer J.; Hardman I.; Shimshack J.; Voorheis J. Disparities in PM2.5 air pollution in the United States. Science. 2020, 369 (6503), 575–578. 10.1126/science.aaz9353. 32732425

[ref58] NY POST. De Blasio Lugs out Giant $15B Check to Stump for Congestion Pricing, 2021. https://nypost.com/2021/07/20/de-blasio-lugs-out-15b-check-to-stump-for-congestion-pricing/.

[ref59] Shah R. U.; Robinson E. S.; Gu P.; et al. Socio-economic disparities in exposure to urban restaurant emissions are larger than for traffic. Environmental Research Letters. 2020, 15 (11), 114039 10.1088/1748-9326/abbc92.

[ref60] Raifman M.; Lambert K. F.; Levy J. I.; Kinney P. L. Mortality Implications of Increased Active Mobility for a Proposed Regional Transportation Emission Cap-and-Invest Program. Journal of Urban Health 2021 98:3. 2021, 98 (3), 315–327. 10.1007/s11524-020-00510-1. PMC7816754 33471280

[ref61] McKelvey W.; Blank J.; Kheirbek I.; Torin B. Using Tracking Data to Promote Environmental Public Health Through Regulatory and Legislative Processes in New York City. Journal of Public Health Management and Practice 2017, 23 (5), S32–S38. 10.1097/PHH.0000000000000619. 28763384

[ref62] Baker K. R.; Amend M.; Penn S. A database for evaluating the InMAP, APEEP, and EASIUR reduced complexity air-quality modeling tools. Data in Brief 2020, 28, 104886 10.1016/j.dib.2019.104886. 31872009 PMC6911961

[ref63] Heo J.; Adams P. J.; Gao H. O. Reduced-form modeling of public health impacts of inorganic PM2.5 and precursor emissions. Atmos. Environ. 2016, 137, 80–89. 10.1016/j.atmosenv.2016.04.026.

[ref64] Tessum C. W.; Paolella D. A.; Chambliss S. E.; Apte J. S.; Hill J. D.; Marshall J. D. PM2.5 polluters disproportionately and systemically affect people of color in the United States. Science Advances 2021, 10.1126/sciadv.abf4491. PMC11426197 33910895

[ref65] Chambliss S.; Pinon C.; Messier K.; et al. Local and Regional-Scale Racial and Ethnic Disparities in Air Pollution Determined by Long-Term Mobile Monitoring. ChemRxiv 2021, 10.26434/CHEMRXIV.14614671.V1. PMC8449331 34493674

[ref66] Paolella D. A.; Tessum C. W.; Adams P. J.; et al. Effect of model spatial resolution on estimates of fine particulate matter exposure and exposure disparities in the United States. ACS Publications. 2018, 5 (7), 436–441. 10.1021/acs.estlett.8b00279.

[ref67] Qian Y.; Li H.; Rosenberg A.; et al. Long-Term Exposure to Low-Level [Formula: see text] and Mortality among the Elderly Population in the Southeastern United States. Environ. Health Perspect. 2021, 129 (12), 127009 10.1289/EHP9044. 34962424 PMC8713651

