# Supplementary material for: ZIP Code-Level Estimation of Air Quality and Health Risk Due to Particulate Matter Pollution in New York City
Source: Environ Sci Technol. 2022 Apr 27;56(11):7119–30. doi: 10.1021/acs.est.1c07325 (PMC9178920; doi:10.1021/acs.est.1c07325)
Supplement: Supplementary file 1 — es1c07325_si_001.pdf [file es1c07325_si_001.pdf]

Supplemental Information for

ZIP code-level estimation of air quality and health risk due to particulate matter pollution in New York City

Komal Shukla[a], Catherine Seppanen[a], Brian Naess[a], Charles Chang[a], David Cooley[b], Andreas Maier[b], Frank Divita[b], Masha Pitiranggon[c], Sarah Johnson[c], Kazuhiko Ito[c], Saravanan Arunachalam[a, *]

a Institute for the Environment, The University of North Carolina at Chapel Hill, Chapel Hill, NC 27599, USA
b Abt Associates, Durham, NC 27703, USA
c New York City Department of Health and Mental Hygiene, Bureau of Environmental Surveillance and Policy, New York, NY 10013, USA

*Corresponding Author: sarav@email.unc.edu

Contents

A) Emissions Data

Table S1: Emission-related data and sources

Table S2: Area sources height of dispersion

Table S3: Stack parameters for waterway sources

Table S4: Area sources a) $PM_{2.5}$ b) NH_3 c) NOx d) SO_2 and e) VOC Tier 1 level NEI 2016 emissions breakdown as spatially allocated in NYC

Table S5: Point sources a) $PM_{2.5}$ b) NH_3 c) NOx d) SO_2 and e) VOC Tier 1 level NEI 2016 emissions breakdown as spatially allocated in NYC

Table S6: Details of emission tiers in ZAPPA

Table S7. Areal extent (in sq. km) of ZCTAs in NYC

Details: A1) Information on web-based integration

A2) Area sources

A3) Point sources

A4) On-road sources

A5) Ships in transit sources

A6) Total $PM_{2.5}$ calculations

Figure S1: County-level NEI 2016 emissions from area sources

Figure S2: County-level NEI 2016 emissions from point sources

Figure S3: County-level NEI 2016 emissions from on-road sources

Figure S4: ZCTA IDs of New York City

B) Health Data and Health Impact Studies

Table S8: Health-related data sources

Table S9: Epidemiological studies used to estimate adverse health impacts of $PM_{2.5}$ (more details in COBRA[1])

C) Results from Illustrative Scenarios

Table S10: Scenario-based declines in annual average ambient $PM_{2.5}$ levels for NYC, associated monetized health benefits (U.S. dollars) in 2016

Figure S5: Avoided health events valuation (in U.S dollars) attributed to $PM_{2.5}$ reduction after implementing policy-based scenario in NYC using ZAPPA-NYC

Figure S6: Scenario (Sc1: reduction from commercial cooking, Sc2: reduction from electrification of passenger cars and school buses, Sc3: congestion pricing in Manhattan, Sc4: reduction from No.4 oil switch in all buildings and Sc5: city-wide sustainable scenario) based a) $PM_{2.5}$ reduction b) avoided mortality c) avoided emergency visits due to asthma d) monetized benefits.

A) Emissions Data

Table S1: Emission-related data and sources

Data Type	Data Source	Year
Emissions (all Tiers)	NEI 2016 v1 Platform (all tiers except below)	2016
Emissions from Fuel Combustion: Commercial/Institutional Oil	NYC DOHMH[2]	2014
Emissions from Off-highway/Aircraft	NEI 2016 v1 Platform: 2020 update[3]	2016
Emissions from Tier combinations (classified as tier 7_1_10: other industrial processes > agriculture, food, & kindred products > commercial cooking) for Grill points	NYC DOHMH[2]	2016
Highway Vehicle activity/on-road emissions	Processed from NEI 2016 v1 Platform and NYC DOHMH[2] (NYCDOHMH data provided a network of road links (individual road segments) with average speed and daily traffic count on each link)	2010/2017

Table S2: Area sources height of dispersion

Tier	Height	Note
#1 (Fuel Combustion: Electric Utility), #2 (Fuel Combustion: Industrial)	44.5 m	
#3 (Fuel Combustion: Commercial and Residential)	5.95 m - 155.9 m	Median building height in the ZIP code
#4 (Chemical & Allied Product Manufacturing), #8 (Solvent Utilization), #9 (Petroleum Storage & Transport), #10 (Waste Disposal & Recycling)	9.1 m	
#12_3 (Off-Highway > Aircraft)	12 m	ZIP codes 11371 (LGA) and 11430 (JFK) only
all other	1 m	

Table S3: Stack parameters for waterway sources

Parameter	Value
stkht	20 m
stkdm	0.8 m
stktmp	555.15 K
stkvel	25 m/s

Table S4 : Area sources a) $PM_{2.5}$ b) NH_3 c) NOx d) SO_2 and e) VOC Tier 1 level NEI 2016 emissions breakdown as spatially allocated in NYC

$PM_{2.5}$ (Tons/year)	Bronx	Kings	New York	Queens	Richmond	Grand Total
Chemical & allied product Manufacturing				0.02		0.02
Fuel combustion: commercial and residential	258.58	536.10	521.62	564.29	154.20	2,034.78
Fuel combustion: electric utility	0.10	0.97	10.27	2.29	0.51	14.15
Fuel combustion: industrial	49.46	199.71	193.69	196.33	10.06	649.24
Highway vehicles	0.00	0.00	0.00	0.00	0.00	0.00
Miscellaneous	240.92	315.08	721.45	497.51	196.56	1,971.52
Off-highway	77.08	169.01	330.09	262.25	45.58	884.00
Other industrial processes	337.66	661.13	446.02	611.97	129.78	2,186.56
Petroleum storage & transport	0.00	0.00	0.00	0.00	0.00	0.00
Solvent utilization	0.00	0.00	0.00	0.02	0.00	0.02
Waste disposal & recycling	11.59	7.89	0.56	1.34	2.65	24.03

NH_3 (Tons/year)	Bronx	Kings	New York	Queens	Richmond	Grand Total
Chemical & allied product manufacturing				0.00		0.00
Fuel Combustion: Commercial and Residential	54.3	133.2	66.3	118.5	29.9	402.2
Fuel combustion: electric utility	0.0	0.0	3.4	0.0	0.0	3.5
Fuel combustion: industrial	4.2	10.6	33.5	10.2	1.4	59.9
Highway vehicles	0.0	0.0	0.0	0.0	0.0	0.0
Miscellaneous	1.8	77.6	2.2	10.2	3.4	95.2
Off-highway	1.6	3.7	6.3	4.0	0.8	16.4
Other industrial processes	0.0	0.0	0.0	0.0	0.0	0.0
Petroleum storage & transport	0.0	0.0	0.0	0.0	0.0	0.0
Solvent utilization	0.0	0.0	0.0	0.0	0.0	0.0
Waste disposal & recycling	7.0	2.8	0.7	16.1	1.7	28.3
NO_x (Tons/Year)	Bronx	Kings	New York	Queens	Richmond	Grand Total
Chemical & allied product manufacturing				0.0		0.0
Fuel Combustion: Commercial and Residential	3,694.6	5,514.5	6,491.4	4,572.9	779.9	21,053.3
Fuel combustion: electric utility	3.2	7.8	132.6	22.1	6.0	171.7
Fuel combustion: industrial	36.6	326.2	441.5	224.4	7.1	1,035.7
Highway vehicles	0.0	0.0	0.0	0.0	0.0	0.0
Miscellaneous	3.4	7.0	3.2	5.3	2.9	21.8
Off-highway	878.1	2,062.8	3,765.6	6,654.9	395.3	13,756.6
Other industrial processes	7.3	25.1	0.5	26.2	7.8	66.9
Petroleum storage & transport	0.0	0.0	0.0	0.0	0.0	0.0
Solvent utilization	0.0	0.0	0.0	0.0	0.0	0.0
Waste disposal & recycling	4.3	21.0	3.5	3.1	8.6	40.5
SO_2 (Tons/Year)	Bronx	Kings	New York	Queens	Richmond	Grand Total
Chemical & allied product manufacturing				0.0		0.0
Fuel Combustion: Commercial and Residential	455.7	204.4	801.0	269.0	32.0	1,762.1
Fuel combustion: electric utility	1.2	0.7	1.6	23.3	0.1	26.9
Fuel combustion: industrial	9.0	28.1	20.5	18.2	0.9	76.8
Highway vehicles	0.0	0.0	0.0	0.0	0.0	0.0
Miscellaneous	0.6	1.9	0.5	0.8	1.1	4.9
Off-highway	2.1	5.1	14.1	572.9	1.7	596.0
Other industrial processes	0.0	0.0	0.0	0.0	0.0	0.0
Petroleum storage & transport	0.0	0.0	0.0	0.0	0.0	0.0
Solvent utilization	0.0	0.0	0.0	0.0	0.0	0.0
Waste disposal & recycling	1.4	16.0	3.2	0.7	0.4	21.7
VOC (Tons/Year)	Bronx	Kings	New York	Queens	Richmond	Grand Total
Chemical & allied product manufacturing				3.8		3.8
Fuel Combustion: Commercial and Residential	143.1	392.8	126.9	470.7	168.1	1,301.5
Fuel combustion: electric utility	0.4	1.2	17.2	0.9	0.4	20.1
Fuel combustion: industrial	10.8	53.2	92.2	58.7	3.3	218.1
Highway vehicles	1.5	2.6	1.3	4.7	1.5	11.5
Miscellaneous	2.1	52.6	1.0	1.1	29.0	85.8
Off-highway	808.9	1,499.1	2,118.2	2,511.7	472.3	7,410.3
Other industrial processes	51.4	108.0	73.2	99.8	22.4	354.8
Petroleum storage & transport	1,226.0	2,479.1	1,528.2	1,730.8	672.5	7,636.6
Solvent utilization	10,057.3	19,279.4	11,081.2	17,760.6	3,541.1	61,719.6
Waste disposal & recycling	59.0	18.1	7.1	102.3	12.2	198.8

Table S5: Point sources a) $PM_{2.5}$ b) NH_3 c) NOx d) SO_2 and e) VOC Tier 1 level NEI 2016 emissions breakdown as spatially allocated in NYC

**$PM_{2.5}$ (Tons/year)**						
Tiers	**Bronx**	**Kings**	**New York**	**Queens**	**Richmond**	**Grand Total**
Fuel Combustion: commercial and residential	3.22	0.00				3.22
Fuel Combustion: electric utility	2.67	11.31	165.75	428.73	47.60	656.07
Fuel Combustion: industrial	0.08	0.89		6.78	0.00	7.75
Petroleum Storage and Transport		0.00	0.00			0.00
Grand total	5.97	12.20	165.75	435.52	47.60	667.04
**NO_x (Tons/year)**						
Tiers	**Bronx**	**Kings**	**New York**	**Queens**	**Richmond**	**Grand Total**
Fuel Combustion: commercial and residential	14.00	0.00				14.00
Fuel Combustion: electric utility	5.34	254.39	1,237.08	2,160.00	539.26	4,196.07
Fuel Combustion: industrial	1.02	76.68		254.71	0.04	332.45
Petroleum Storage and Transport		0.00	0.00			0.00
Grand total	20.37	331.07	1,237.08	2,414.70	539.30	4,542.52
**NH_3 (Tons/year)**						
Tiers	**Bronx**	**Kings**	**New York**	**Queens**	**Richmond**	**Grand Total**
Fuel Combustion: commercial and residential	1.64	0.00				1.64
Fuel Combustion: electric utility	2.37	6.63	49.97	84.56	22.64	166.17
Fuel Combustion: industrial	0.01	35.41		0.09	0.00	35.51
Petroleum Storage and Transport		0.08	0.00			0.08
**SO_2 (Tons/year)**						
Tiers	**Bronx**	**Kings**	**New York**	**Queens**	**Richmond**	**Grand Total**
Fuel Combustion: commercial and residential	0.25	0.00				0.25
Fuel Combustion: electric utility	0.32	3.35	119.58	136.56	3.81	263.63
Fuel Combustion: industrial	0.06	6.39		3.07	0.00	9.53
Petroleum Storage and Transport		0.00	0.00			0.00
**VOC (Tons/year)**						
Tiers	**Bronx**	**Kings**	**New York**	**Queens**	**Richmond**	**Grand Total**
Fuel Combustion: commercial and residential	2.20	0.00				2.20
Fuel Combustion: electric utility	1.01	4.16	163.88	215.63	44.73	429.41
Fuel Combustion: industrial	0.09	0.42		28.47	0.00	28.99
Petroleum Storage and Transport		0.00	0.15			0.15

Table S6: Details of emission tiers in ZAPPA

Tier 1	Tier 1 Name	Tier 2	Tier 2 Name	Tier 3	Tier 3 Name
1	Fuel Combustion: Electric Utility	2	Oil		
		3	Gas		
		5	Internal Combustion		
2	Fuel Combustion: Industrial	2	Oil		
		3	Gas		
		4	Other		
		5	Internal Combustion		
3	Fuel Combustion: Commercial and Residential	2	Commercial/Institutional Oil		
		3	Commercial/Institutional Gas		
		4	Misc. Fuel Comb. (Except Residential)		
		5	Residential Wood		
		6	Residential Other	1	Distillate Oil
				2	Natural Gas
				99	Other
7	Other Industrial Processes	1	Agriculture, Food, & Kindred Products	10	Commercial Cooking
				99	Other
		5	Mineral Products		
		6	Machinery Products		
		99	Miscellaneous Industrial Processes		
8	Solvent Utilization	1	Degreasing		
		2	Graphic Arts		
		3	Dry Cleaning		
		4	Surface Coating		
		6	Nonindustrial		
9	Petroleum Storage & Transport	1	Bulk Terminals & Plants		
		2	Petroleum & Petroleum Product Storage		
		3	Petroleum & Petroleum Product Transport		
		4	Service Stations: Stage I		
		5	Service Stations: Stage II		
		6	Service Stations: Breathing & Emptying		
		7	Organic Chemical Storage		
		9	Inorganic Chemical Storage		
10	Waste Disposal & Recycling	1	Incineration		
		2	Open Burning		
		3	Publicly Owned Treatment Works		
		6	Landfills		
		7	Other		
11	Highway Vehicles	11	Diesel Fuel	99	Refueling
		14	Gasoline	99	Refueling
12	Off-Highway	1	Non-Road Gasoline	2	Construction
				99	All Other

		2	Non-Road Diesel	2	Construction
				99	All Other
		3	Aircraft		
		4	Marine Vessels	2	Diesel
		5	Railroads		
		6	Other		
14	Miscellaneous	1	Agriculture & Forestry		
		2	Other Combustion		
		5	Health Services		
		7	Other Fugitive Dust		

A1) Information on web-based integration: The ZAPPA's easy-to-use GUI allows the user to compare the changes in total $PM_{2.5}$ concentration and respective co-benefit achieved in any ZCTA in NYC. The visual outputs (and data downloads) from ZAPPA are changes in concentrations, health benefits and their associated monetized values. Each of these outputs can be visualized for the whole city, a subset of ZCTAs or even individual ZCTAs. The model outputs can also be downloaded as text or shapefiles for offline use.

A2) Area sources: Based on the augmented NEI emissions inventories described above, the NYC region contains significant amounts of $PM_{2.5}$ (~7,764 tpy (tons/year)), NH_3 (605.4 tpy), NOx (36,146.4 tpy), SO_2 (2,488.4 tpy), and VOC (78,960.8 tpy (Figure 2(a) in manuscript and Figure S1 here). The highest amount of $PM_{2.5}$ emissions in NYC are from the SCC tier *other industrial processes* (a total of 2,186 tpy, out of which 2,173 tpy comes from sub-tier *agriculture, food, & kindred products*; which is mainly commercial cooking using grills) followed by the tier *Fuel combustion: commercial and residential* (a total of 2,034 tpy, out of which 870 tpy is released from sub-tier from *residential other*, 590 tpy from *residential wood* and 285 tpy from *miscellaneous fuel combustion (except residential)* and lastly from the miscellaneous emission tier which mainly comprises of fugitive dust (1,969 tpy).

A3) Point sources: 72 individual point-based emissions are from power generating plants (*fuel combustion: electric utility*) in the city, and they are non-uniformly distributed across all 192 ZCTAs. The emissions in tpy are as follows: $PM_{2.5}$ (667), NOx (4,543), NH_3 (203), SO_2 (237), and VOC (460). Queen's borough is responsible for the most point source-related emissions (Figure 2(b) in manuscript and Figure S2 here.

A4) On-road sources: With 24,881 road links in NYC, $PM_{2.5}$ emissions are 913 tpy, NH_3 515 tpy, NOx 14,520 tpy, SO_2 222 tpy, SOA 44 tpy (Figure 2c in manuscript). Queens (road links : 8,021, length : 1,118 km, AADT: 208 M, and $PM_{2.5}$ emissions :346 tpy) has the highest amount of on-road activity followed by Kings (road links : 5,221, length : 598 km, AADT: 104 M, and $PM_{2.5}$ emissions :217.6 tpy), Manhattan (road links : 3,672, length : 504 km, AADT: 85.6 M, and $PM_{2.5}$ emissions :172 tpy), Bronx (road links : 5,221, length : 598 km, AADT: 82.7 M, and $PM_{2.5}$ emissions :117 tpy), and Staten Island (road links : 2,912, length : 388 km, AADT: 39.5 M, and $PM_{2.5}$ emissions :61 tpy).

A5) Ships in transit emissions: Waterway emissions were distributed across the rivers, bays, and sounds surrounding NYC (Figure 2d). These regions include East River, Harlem River, Long Island sound, Lower Bay, Lower Hudson River, Upper Bay and Upper Hudson River.

A6) Total $PM_{2.5}$ calculations: Total $PM_{2.5}$ is a summation of locally-emitted primary $PM_{2.5}$ (from C-TOOLS), non-local (transported) primary $PM_{2.5}$ (from COBRA), and secondary $PM_{2.5}$. Secondary $PM_{2.5}$ is taken as the sum of SOA, ammonium bisulfate (NH_4HSO_4), ammonium sulfate ($(NH_4)_2SO_4$), and ammonium nitrate (NH_4NO_3). The formation of latter three secondary components is based on the reduced form treatment of chemistry in COBRA. SOA is taken as the sum of locally-produced SOA (from C-TOOLS) and transported SOA (from COBRA). The transported concentrations of primary $PM_{2.5}$ and SOA are generated by running COBRA with zero local emissions; the resulting

primary $PM_{2.5}$ and SOA concentrations are taken to be the transported fraction. The total $PM_{2.5}$ calculated by the integrated tool for base year 2016 is stored in the database as the baseline estimate.

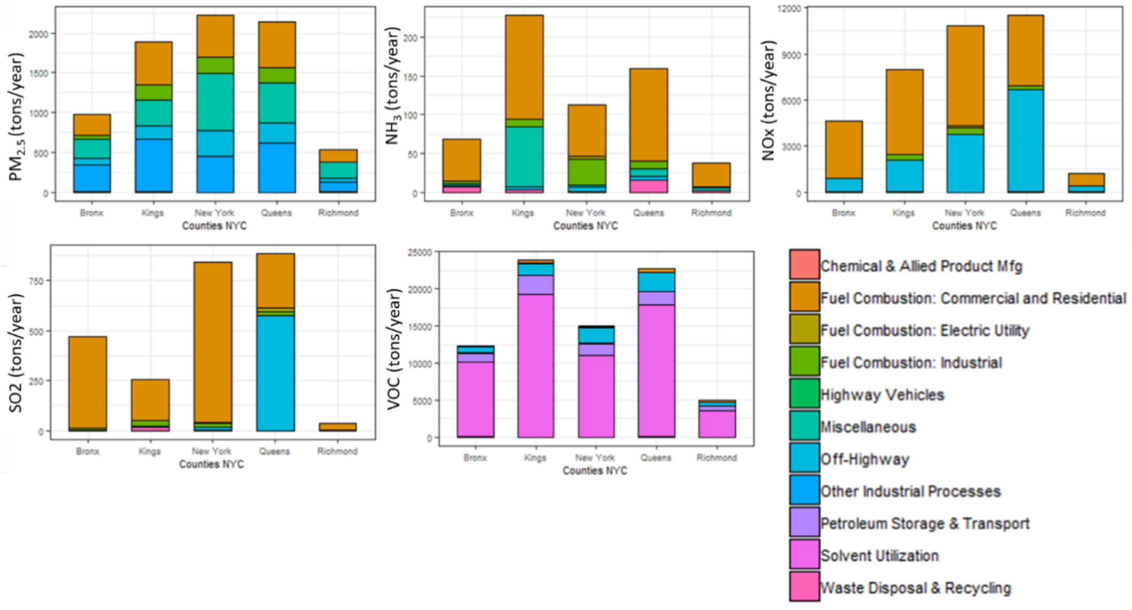

Figure S1: County-level NEI 2016 emissions from area sources

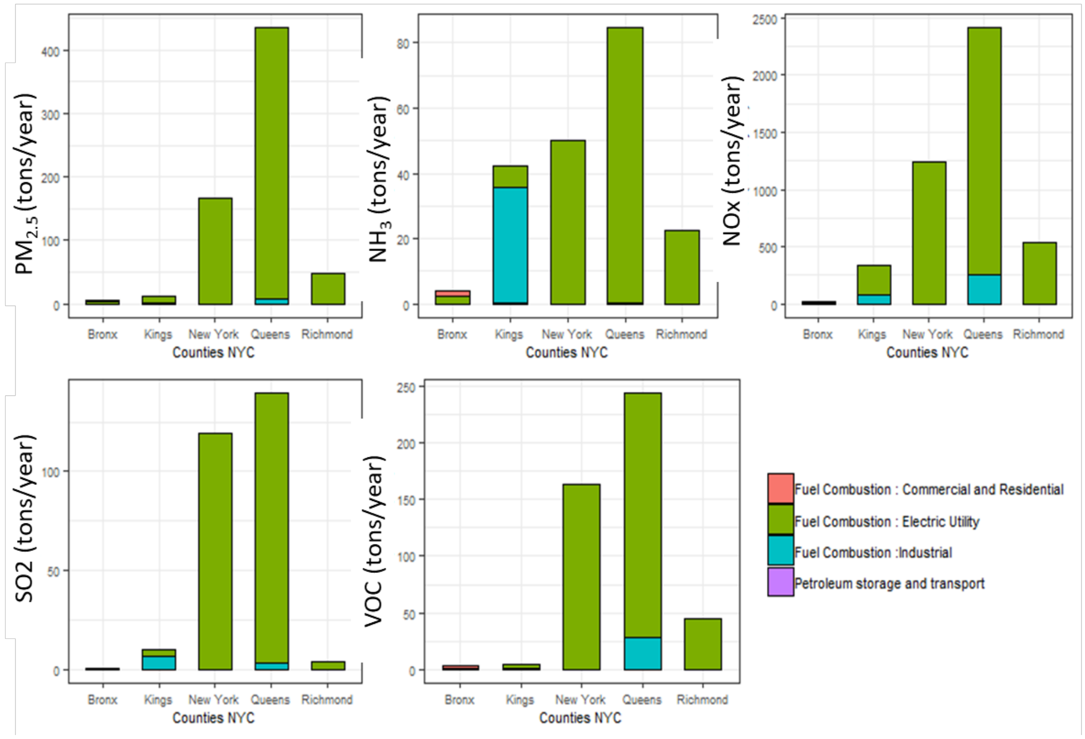

Figure S2: County-level NEI 2016 emissions from point sources

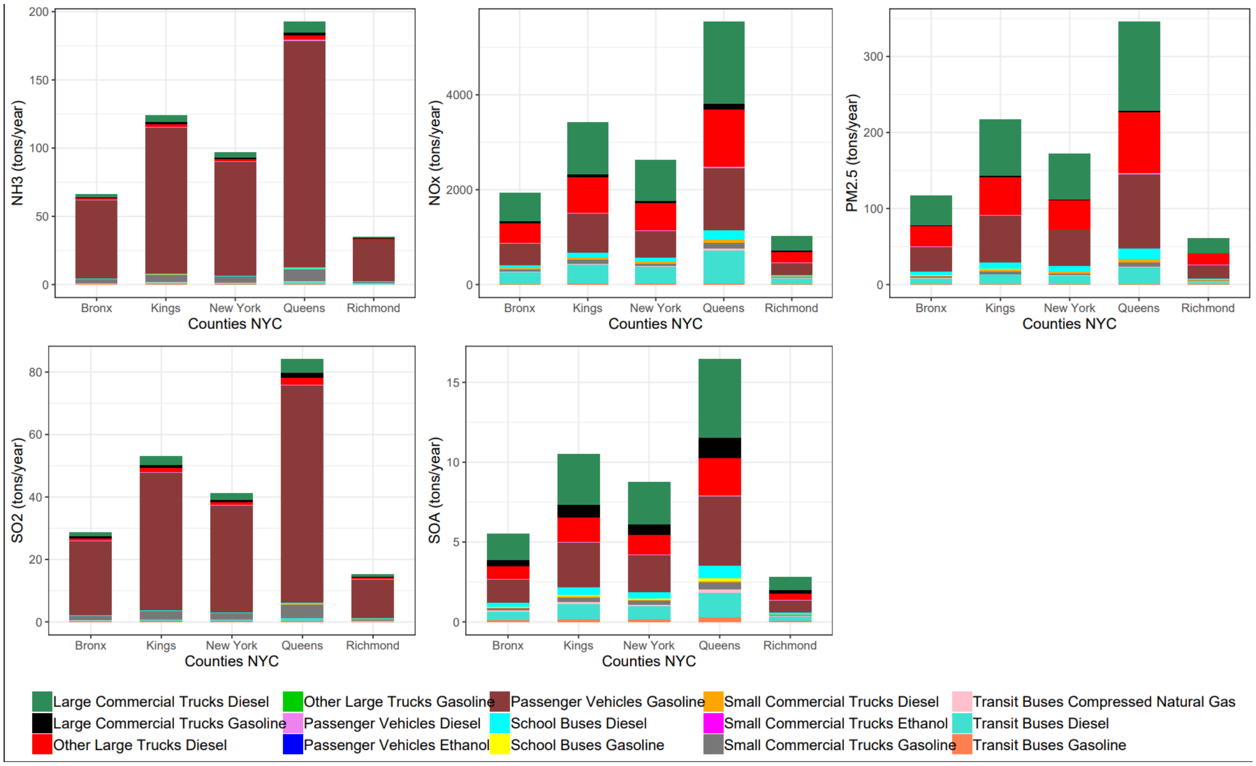

Figure S3: County-level NEI 2016 emissions from on-road sources

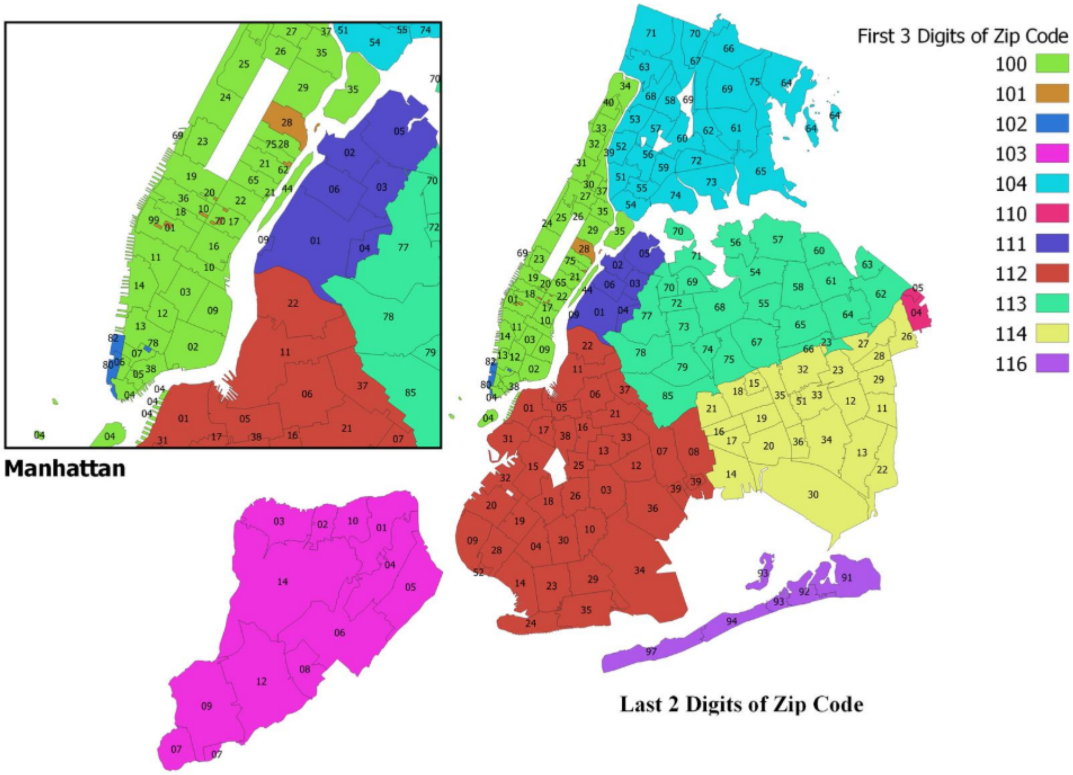

Figure S4 : ZCTA IDs of New York City

Table S7. Areal extent (in sq. km) of ZCTAs in NYC

FIPS Code	FIPS Name	Average	Maximum	Minimum	Max ZCTA ID	Min ZCTA ID
36005	Bronx	2.4	8.9	0.0005	10465	10464
36047	Kings	4.5	21.7	0.0039	11234	11211
36061	New York	0.8	2.9	0.0066	10034	10004
36081	Queens	4.1	19.1	0.0191	11430	11103
36085	Richmond	10.8	37.3	0.0266	10314	10301

B) Health impact data from COBRA and other epidemiological studies

Table S8 : Health-related data and sources

Data Type	Data Source	Year
ZIP code-level health incidence data	NYC DOHMH[3]	2015-2017
ZIP code-level population data	NYC DOHMH[3]	2015-2017
C-R functions (all except below)	COBRA[1]	2016
C-R functions for "Emergency Department Visits, Asthma" and "Hospital Admits, Cardio"	NYC DOHMH[3]	2015-2017
County-level health incidence data	COBRA[1]	2016
County-level population data	COBRA[1]	2016

Table S9: Epidemiological studies used to estimate adverse health impacts of $PM_{2.5}$ (more details in COBRA[1])

Endpoint	Start and End age	Function	Study
Acute Bronchitis*	8-12	$\left(1-\frac{1}{(1-Inc)*exp^{Beta*\Delta Q}+Inc}\right)*Inc*Pop$	Dockery et al.,1996
Acute Myocardial Infarction, Nonfatal (high)	18-99	$\left(1-\frac{1}{(1-Inc*A)*exp^{Beta*\Delta Q}+Inc*A}\right)*Inc*A*Pop$	Peters et al.,2001
Acute Myocardial Infarction, Nonfatal (low)	0-99		Pope et al.,2006
Acute Myocardial Infarction, Nonfatal (low)	0-99		Sullivan et al.,2005
Acute Myocardial Infarction, Nonfatal (low)	0-99		Zanobetti and Schwartz,2006
Acute Myocardial Infarction, Nonfatal (low)	0-99	$\left(1-(\frac{1}{exp^{(Beta*\Delta Q)}})\right)*Inc*Pop*A$	Zanobetti et al.,2009
Asthma Exacerbation, Cough	6-17	$\left(A-(\frac{A}{(1-A)exp^{(Beta*\Delta Q)}+A})\right)Pop*B$	Mar et al.,2004
Asthma Exacerbation, Cough	6-17	$\left(1-(\frac{1}{(1-A)exp^{(Beta*\Delta Q)}+A})\right)*A*Pop*B$	Ostro et al.,2001
Asthma Exacerbation, Shortness of Breath	6-17		Ostro et al.,2001
Asthma Exacerbation, Wheeze	6-17		Ostro et al.,2001
Asthma Exacerbation, Cough	18-18		Ostro et al.,2001
Asthma Exacerbation, Shortness of Breath	18-18		Ostro et al.,2001
Asthma Exacerbation, Wheeze	18-18		Ostro et al.,2001
Asthma Exacerbation, Shortness of Breath	18-18	$\left(A-(\frac{A}{(1-A)exp^{(Beta*\Delta Q)}+A})\right)Pop*B$	Mar et al.,2004
Asthma Exacerbation, Cough	18-18		Mar et al.,2004
Asthma Exacerbation, Shortness of Breath	6-17		Mar et al.,2004
Emergency Room Visits, Asthma	0-99	$\left(1-exp^{(-Beta*\Delta Q)}\right)*Inc*Pop$	Mar et al.,2010
Emergency Room Visits, Asthma	0-99		Slaughter et al.,2005
Emergency Room Visits, Asthma	0-99	$\left(Inc-(\frac{Inc}{(1-Inc)exp^{(Beta*\Delta Q)}+Inc})\right)*Pop$	Glad et al.,2012
HA, All Cardiovascular (less Myocardial Infarctions)	18-64	$\left(1-(\frac{1}{exp^{(Beta*\Delta Q)}})\right)*Inc*Pop$	Moolgavkar,2000
HA, All Cardiovascular (less Myocardial Infarctions)	65-99		Bell et al.,2008
HA, All Cardiovascular (less Myocardial Infarctions)	65-99		Peng et al.,2008
HA, All Cardiovascular (less Myocardial Infarctions)	65-99		Peng et al.,2009
HA, All Cardiovascular (less Myocardial Infarctions)	65-99		Zanobetti et al,2009
HA, All Respiratory	65-99		Zanobetti et al,2009
HA, All Respiratory	65-99		Kloog et al.,2012
HA, Asthma	0-17		Babin et al.,2007
HA, Asthma	0-17		Sheppard,2003
HA, Chronic Lung Disease	18-64		Moolgavkar,2000
Lower Respiratory Symptoms	7-14	$\left(1-\frac{1}{(1-A)*exp^{Beta*\Delta Q}+A}\right)*A*Pop$	Schwartz and Neas,2000
Minor Restricted Activity Days	18-64	$\left(1-(\frac{1}{exp^{(Beta*\Delta Q)}})\right)*Pop*A$	Ostro and Rothschild,1989

Mortality, All Cause (low)*	30-99	$\left(1-(\frac{1}{exp^{(Beta*\Delta Q)}})\right)*Inc*A$	Krewski et al.,2009
Mortality, All Cause (high)*	25-99	$\left(1-(\frac{1}{exp^{(Beta*\Delta Q)}})\right)*Inc*Pop*A$	Lepeule et al.,2012
Infant Mortality*	0-0	$\left(1-\frac{1}{(1-Inc)*exp^{Beta*\Delta Q}+Inc}\right)*Inc*Pop$	Woodruff et al.,1997
Upper Respiratory Symptoms	9-11	$\left(1-(\frac{1}{(1-A)exp^{(Beta*\Delta Q)}+A})\right)*A*Pop*B$	Pope et al.,1991
Work Loss Days	18-64	$\left(1-(\frac{1}{exp^{(Beta*\Delta Q)}})\right)*Inc*Pop$	Ostro,1987
Emergency Department Visits, Asthma	0-99		Ito et al.,2007
HA, All Cardiovascular	40-99		Ito et al.,2011

C) Extended results from various illustrative scenarios

Table S10: Scenario based declines in Annual Average Ambient $PM_{2.5}$ Levels for NYC, associated monetized health benefits (U.S. dollars) in 2016

Avoided Event | Scenario	Sc1 Emission reduction from grill-based cooking	Sc2 Emission reduction from electrification of passenger cars and school buses	Sc3 Emission reduction from congestion pricing in Manhattan	Sc4 Emission reduction from No.4 oil switch in all buildings	Sc5 City-wide sustainable scenario (Sc1+Sc2+Sc3 +Sc4)
Total Health Benefits (low estimate) ($)	$1,563,391,035	$807,465,791	$27,709,410	$69,558,095	$2,288,847,824
Total Health Benefits (high estimate) ($)	$3,507,985,575	$1,814,582,146	$62,209,840	$156,390,624	$5,130,041,867
Mortality (low estimate) ($)	$1,525,757,094	$788,398,445	$27,103,778	$67,932,060	$2,234,229,916
Mortality (high estimate) ($)	$3,450,411,436	$1,785,132,107	$61,285,198	$153,950,856	$5,046,467,524
Infant Mortality ($)	$9,783,992	$5,114,070	$91,582	$425,364	$14,233,084
Nonfatal Heart Attacks (low estimate) ($)	$2,425,573	$1,257,087	$38,515	$98,183	$3,534,146
Nonfatal Heart Attacks (high estimate) ($)	$22,365,772	$11,639,781	$357,524	$911,917	$32,490,582
Hospital Admits, All Respiratory ($)	$1,142,376	$584,560	$14,471	$55,021	$1,666,477
Hospital Admits, Cardiovascular ($)	$1,687,873	$895,163	$22,944	$73,610	$2,479,087
Acute Bronchitis ($)	$143,418	$74,947	$1,489	$6,087	$207,739
Upper Respiratory Symptoms ($)	$180,584	$94,053	$1,853	$7,613	$262,391
Lower Respiratory Symptoms ($)	$80,268	$41,942	$825	$3,401	$116,373
Emergency Room Visits, Asthma ($)	$132,322	$67,390	$1,328	$6,901	$192,914
Minor Restricted Activity Days ($)	$15,537,184	$7,708,299	$305,942	$669,746	$22,488,976
Work Loss Days ($)	$6,195,832	$3,061,186	$123,245	$266,330	$8,965,294
Asthma Exacerbation ($)	$324,517	$168,648	$3,438	$13,778	$471,425

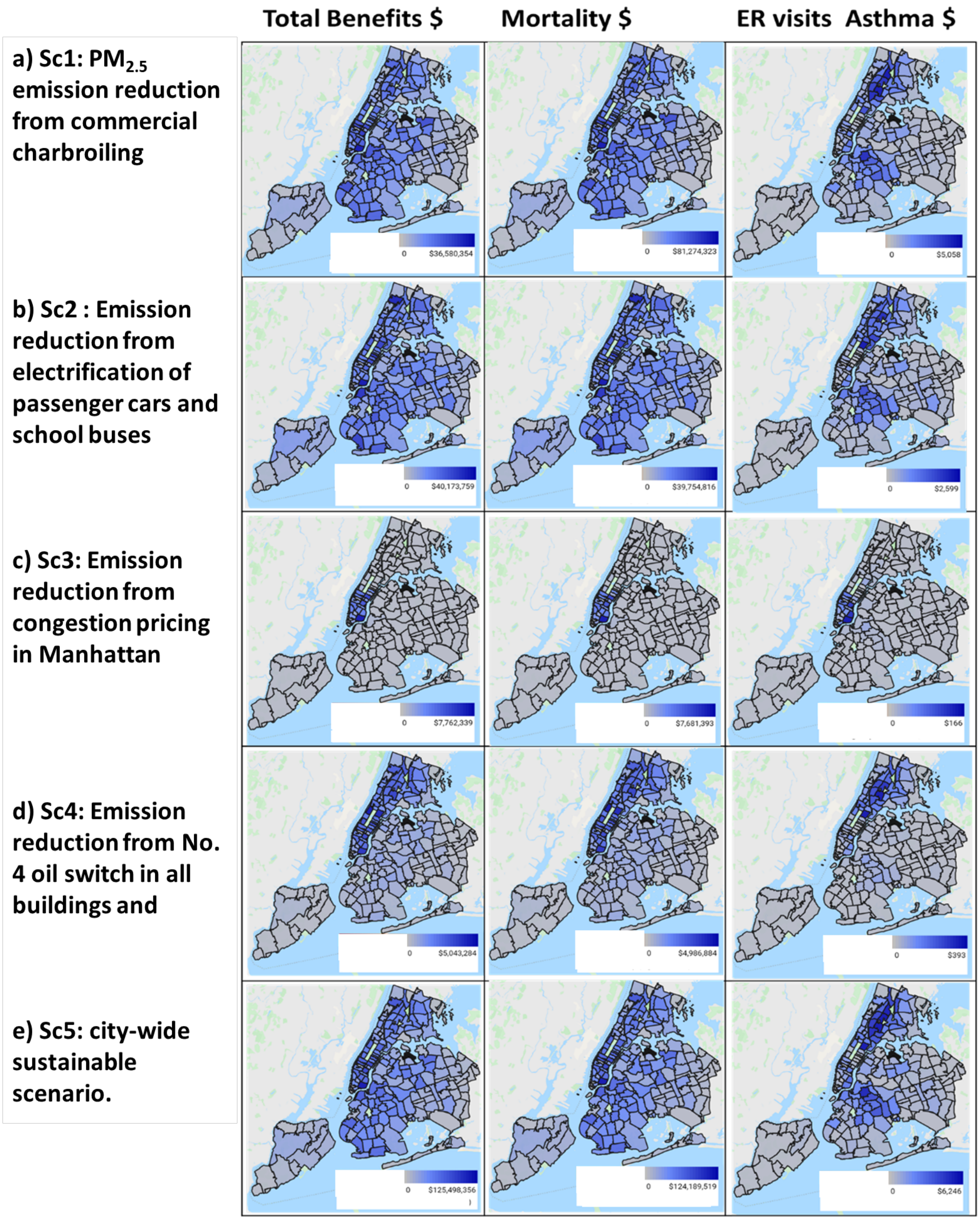

Figure S5: Avoided health events valuation (in U.S dollars) attributed to $PM_{2.5}$ reduction after implementing policy-based scenario in NYC using ZAPPA-NYC

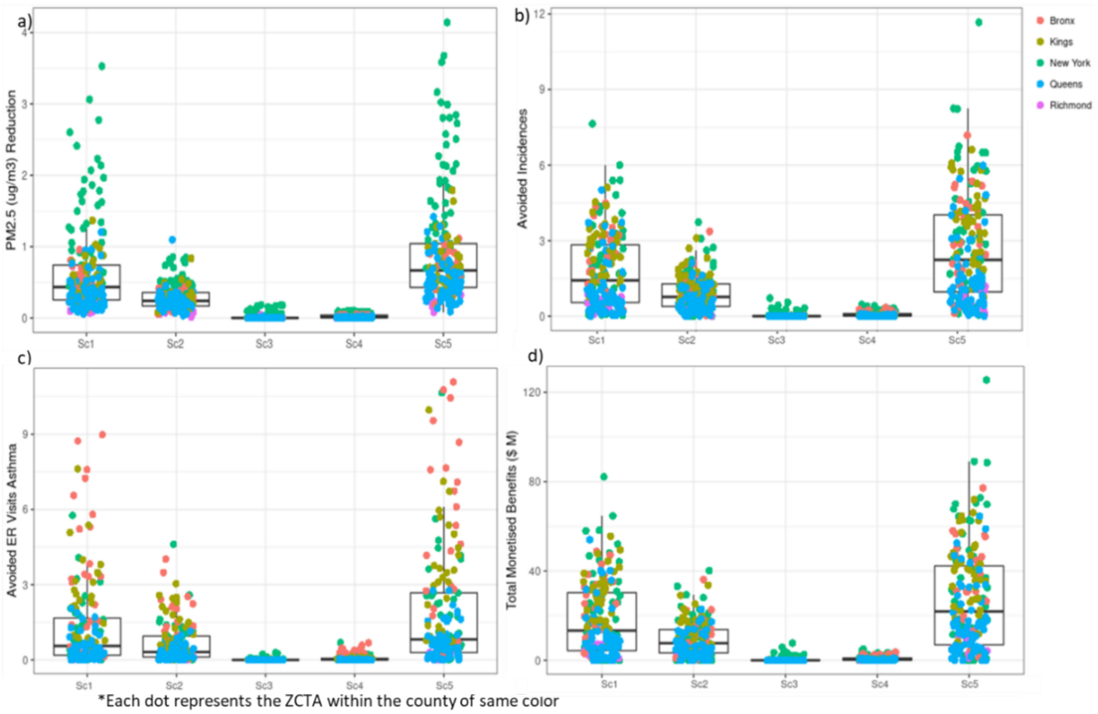

Figure S6: Scenario (Sc1: reduction from commercial cooking, Sc2: reduction from electrification of passenger cars and school buses, Sc3: congestion pricing in Manhattan, Sc4: reduction from No.4 oil switch in all buildings and Sc5: city-wide sustainable scenario) based a) $PM_{2.5}$ reduction b) avoided mortality c) avoided emergency visits due to asthma d) monetized benefits.

References

1. U.S. EPA. User's Manual for the Co-benefits Risk Assessment (COBRA) Screening Model Version: 2.614 . https://www.epa.gov/cobra.

2. NYC DOHMH. New York City Department of Health and Mental Hygiene. Environment & Health Data Portal. http://a816-dohbesp.nyc.gov/IndicatorPublic/Subtopic.aspx?theme_code=2,3&subtopic_id=103.

3. National Emission Inventory UE. NEI 2016. https://www.epa.gov/air-emissions-inventories/2017-national-emissions-inventory-nei-data.
